# Deeply functional identification of *TCS1* alleles provides efficient technical paths for low-caffeine breeding of tea plants

**DOI:** 10.1093/hr/uhac279

**Published:** 2022-12-21

**Authors:** Yi Wang, Yu-Fei Liu, Meng-Yuan Wei, Chen-Yu Zhang, Jie-Dan Chen, Ming-Zhe Yao, Liang Chen, Ji-Qiang Jin

**Affiliations:** Key Laboratory of Biology, Genetics and Breeding of Special Economic Animals and Plants, Ministry of Agriculture and Rural Affairs; Tea Research Institute of the Chinese Academy of Agricultural Sciences, Hangzhou 310008, China; Key Laboratory of Biology, Genetics and Breeding of Special Economic Animals and Plants, Ministry of Agriculture and Rural Affairs; Tea Research Institute of the Chinese Academy of Agricultural Sciences, Hangzhou 310008, China; Yunnan Provincial Key Laboratory of Tea Science, Tea Research Institute, Yunnan Academy of Agricultural Sciences, 2 Jingnan Road, Menghai, Yunnan 666201, China; Key Laboratory of Biology, Genetics and Breeding of Special Economic Animals and Plants, Ministry of Agriculture and Rural Affairs; Tea Research Institute of the Chinese Academy of Agricultural Sciences, Hangzhou 310008, China; Key Laboratory of Biology, Genetics and Breeding of Special Economic Animals and Plants, Ministry of Agriculture and Rural Affairs; Tea Research Institute of the Chinese Academy of Agricultural Sciences, Hangzhou 310008, China; Key Laboratory of Biology, Genetics and Breeding of Special Economic Animals and Plants, Ministry of Agriculture and Rural Affairs; Tea Research Institute of the Chinese Academy of Agricultural Sciences, Hangzhou 310008, China; Key Laboratory of Biology, Genetics and Breeding of Special Economic Animals and Plants, Ministry of Agriculture and Rural Affairs; Tea Research Institute of the Chinese Academy of Agricultural Sciences, Hangzhou 310008, China; Key Laboratory of Biology, Genetics and Breeding of Special Economic Animals and Plants, Ministry of Agriculture and Rural Affairs; Tea Research Institute of the Chinese Academy of Agricultural Sciences, Hangzhou 310008, China; Key Laboratory of Biology, Genetics and Breeding of Special Economic Animals and Plants, Ministry of Agriculture and Rural Affairs; Tea Research Institute of the Chinese Academy of Agricultural Sciences, Hangzhou 310008, China

## Abstract

Caffeine is an important functional component in tea, which has the effect of excitement and nerve stimulation, but excessive intake can cause insomnia and dysphoria. Therefore, the production of tea with low-caffeine content can meet the consumption needs of certain people. Here, in addition to the previous alleles of the tea caffeine synthase (TCS1) gene, a new allele (*TCS1h*) from tea germplasms was identified. Results of *in vitro* activity analysis showed that *TCS1h* had both theobromine synthase (TS) and caffeine synthase (CS) activities. Site-directed mutagenesis experiments of *TCS1a*, *TCS1c*, and *TCS1h* demonstrated that apart from the 225th amino acid residue, the 269th amino acid also determined the CS activity. GUS histochemical analysis and dual-luciferase assay indicated the low promoter activity of *TCS1e* and *TCS1f.* In parallel, insertion and deletion mutations in large fragments of alleles and experiments of site-directed mutagenesis identified a key *cis*-acting element (G-box). Furthermore, it was found that the contents of purine alkaloids were related to the expression of corresponding functional genes and alleles, and the absence or presence and level of gene expression determined the content of purine alkaloids in tea plants to a certain extent. In summary, we concluded *TCS1* alleles into three types with different functions and proposed a strategy to effectively enhance low-caffeine tea germplasms in breeding practices. This research provided an applicable technical avenue for accelerating the cultivation of specific low-caffeine tea plants.

## Introduction

Tea plant (*Camellia sinensis* (L.) O. Kuntze) is an economical beverage plant, which has been cultivated in more than 60 countries [1]. Tea, which is processed from young shoots, has become the second most popular beverage after water. Its popularity is closely correlated with its diverse biochemical constituents, including theanine, catechins, and purine alkaloids. Among them, purine alkaloids (3.0%–6.0% dry weight), as the characteristic secondary metabolites of tea, primarily contain caffeine (1,3,7-trimethylxanthine), theobromine (3,7-dimethylxanthine), and occasionally theacrine (1,3,7,9-tetramethyluric acid) with a bitter taste and resistance to some biotic and abiotic stresses [2, 3]. However, the types and concentrations of the above-mentioned purine alkaloids vary greatly among tea plants [2, 4]. Caffeine (2.5%–5.0%) is the main purine alkaloid in the cultivated *C. sinensis* (L.) O. Kuntze and some *C. taliensis* (W. W. Smith) Melchior. Only a few special tea plants in ‘cocoa tea’ (CCT) (*C. ptilophyll* Chang, a caffeine-free tea plant from Guangdong, China) and ‘Kucha’ (i.e. bitter tea) contain high theobromine or theacrine, respectively [5, 6].

Caffeine is an important biochemical component affecting the quality and function of tea. It exhibits not only antioxidant [7] and anti-inflammatory [8] activities, but also protection against various diseases, such as cardiovascular diseases [9], obesity [10], and cancers [11]. However, chronic or excessive caffeine consumption could lead to addiction, insomnia [12], migraines [13], and other side effects [14]. In addition, children, adolescents, pregnant women, and those sensitive to caffeine must limit or reduce their intake to avoid adverse effects [15]. For pregnant women, in particular, prenatal caffeine exposure could affect offspring development, induce cognitive impairment, and increase susceptibility to disease in adulthood [14]. Therefore, the creation of low-caffeine tea (caffeine <1.0%) has attracted extensive attention. Multiple industrial processes such as solvent extraction, supercritical fluid extraction with carbon dioxide (CO_2_), microbial degradation, and enzymatic degradation were adopted in de-caffeinate [16]. However, breeding low-caffeine tea cultivars remains an efficient and safe method compared with industrial processes; some progress has been made in the breeding of low-caffeine tea plants [17]. A functional marker for caffeine-free tea plants had been developed for low-caffeine breeding, while it needed to be detected by gene sequencing [18]. Moreover, breeding has been greatly hindered because of the difficulties in breeding and poor adaptability of special tea plants, such as CCT. Therefore, investigating the differential enrichment mechanisms of purine alkaloids among various tea plants will facilitate the cultivation of low-caffeine tea cultivars.

The core synthetic pathway of caffeine involves three methylation steps and one de-nucleoside reaction. *N*-methyltransferases (NMTs) play a key role in regulating the biosynthesis of caffeine in plants [19]. Notably, *CsNMT17* (tea caffeine synthase gene, *TCS1*) is expressed with higher expression levels than other NMT genes, suggesting its predominant roles in caffeine biosynthesis [20]. TCS1 is the most critical NMT enzyme in the caffeine biosynthesis pathway, which catalyzes the methylation of *N*-3 and *N*-1 [6, 21]. The first NMT gene to be cloned was *TCS1* [22], after which five homologous genes of *TCSs* (*TCS2–6*) and seven allelic variations of *TCS1* (a, b, c, d, e, f, g) were isolated from tea plants [21, 23]. In addition, *TCS1i* has been found in a unique tea plant, namely, ‘Hongyacha’ (HYC) [24]. Although tea plants had multiple NMT genes related to caffeine synthesis, many of them had no function or low expression [1, 25, 26]. Several allelic variations with markedly different enzyme activities or expression levels are found in a few specific tea germplasms [21]. NMTs involving purine alkaloid biosynthesis from different genera evolve independently at least two times [27–29]. It made NMTs of different genera less similar and also generated a diversity of variations in *TCS1* sequences resulting in diverse function of *TCS1*. Previous studies already showed that the 225th amino acid residue of *TCS1a* plays an important role in substrate specificity, and its mutation directly determined whether it had CS activity or not [21, 30]. However, *TCS1* alleles are not sufficiently investigated at present, and the expression regulation and genetic mechanism of different *TCS1* alleles remain unclear, thereby limiting the effective utilization of specific tea germplasms. For example, in thepractice of crossbreeding, practical difficulties such as parents not meeting the flowering period, incompatibility, and poor adaptability are often encountered. Therefore, finding more tea plants carrying specific *TCS1* alleles could enhance the breeding success rate.

In this study, we systematically revealed *TCS1* allele variations. Using the TCS1-InDel marker, a novel allele was identified from 673 tea accessions. The CDS sequence of different alleles was isolated to construct the recombinant protein and study the new key active sites of caffeine synthase (CS); the promoter activities of the genes were determined by *β*-glucuronidase (GUS) staining of stable transgenic *Arabidopsis thaliana* and the dual-luciferase reporter assay system. By performing site-directed mutagenesis experiments, G-Box was found to be a key *cis-*acting element affecting the promoter activities of different *TCS1* alleles. Based on the breeding practice of low caffeine in the past 10 years, efficiently innovating new germplasms with low caffeine was proposed. These results will promote the understanding of caffeine biosynthesis in tea plants, revise previous conclusions and innovate specific tea germplasms through rare allelic variations.

## Results

### Screening and analysis of novel alleles of *TCS1* in tea resources

Different genetic background of low-caffeine resources were newly discovered in Yunnan and its adjacent region, which indicates that there may be new *TCS1* allelic variations in tea resources [31, 32]. To deeply excavate more novel *TCS1* alleles, genomic DNA of 673 accessions of tea genetic resources was collected from Yunnan and the National Germplasm Hangzhou Tea Repository in Zhejiang. The previously acquired TCS1-InDel marker [21] was used to find tea plants carrying different rare *TCS1* alleles **(**Fig. S1, see online supplementary material**)**. Consequently, a novel allele of 338 bp, namely, *TCS1h*, was isolated from a few special tea plants **(**Tables S1 and S2, see online supplementary material**)**. Until now, combined with previous studies, a total of nine *TCS1* alleles with evident sequence differences have been found in natural tea plants.

**Figure 1:**
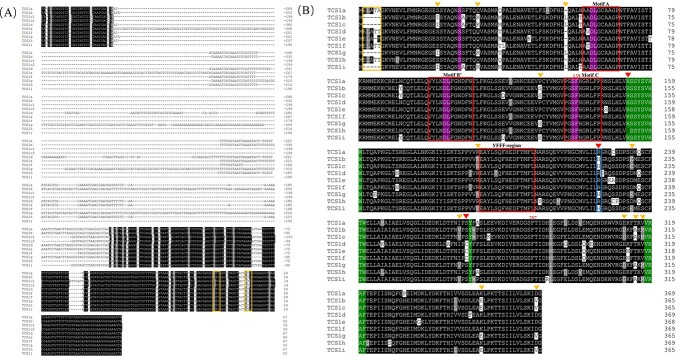
Comparison of *TCS1* allele promoter sequences and amino acid sequences. **A** Comparison of promoter sequences between *TCS1* alleles. The yellow box marks the initiation codon (ATG). **B** Comparison of amino acid sequences of TCS1h and other TCS1s. The proposed SAM-binding motifs (A, B′, and C) and conserved region that are nominated as the ‘YFFF-region’ are shown in red empty boxes. The amino acid residues are marked in a blue empty box, and they played a critical role in substrate recognition. The nominated amino acids in substrate binding are indicated by purple (SAM) and red (methyl acceptor) boxes. The TCS1b, TCS1c, TCS1g, and TCS1i variant sites compared with TCS1a, TCS1d, TCS1e, TCS1f, and TCS1h are indicated by a brownish yellow triangle. The important mutation sites are marked with red triangles. The TCS1b, TCS1c, TCS1g, and TCS1i deletion motif ‘ELATA’ is marked with a yellow empty box.

Further analysing the sequence differences, the sequence alignment of the 5′ upstream regulatory region showed that the initiation codon ‘ATG’ of *TCS1g*, *TCS1i*, *TCS1b*, and *TCS1c* was 15 bp later than that of *TCS1h*, *TCS1a*, *TCS1d*, *TCS1e*, and *TCS1f***(**Fig. 1A; Table S3; see online supplementary material**)**. The amino acid sequence similarity of TCS1h with TCS1a, TCS1d, TCS1e, and TCS1f was more than 92.12% **(**Table S4, see online supplementary material**)**. TCS1a, TCS1d, TCS1e, TCS1f, and TCS1h compared to TCS1b, TCS1c, TCS1g, and TCS1i had 13 amino acid residue substitutions. We also found that the amino acid at the 225th position of five alleles, including TCS1a, TCS1d, TCS1e, TCS1f, and TCS1h, was arginine (Arg), whereas the corresponding amino acid of the other four alleles was histidine (His) **(**Fig. 1B**)**.

### Functional identification of proteins encoded by different *TCS1* alleles

**Figure 2:**
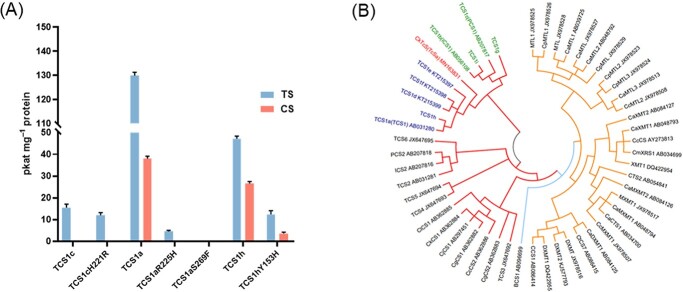
Functional studies of proteins encoded by different *TCS1* alleles and comparison of phylogenetic trees of *N*-methyltransferases. **A** Activity assay results of TCS1 recombinant proteins (*n* = 3). **B** Phylogenetic tree of *N*-methyltransferase coding region sequences related to purine alkaloids in *Camellia* (red), *Coffea* (yellow), and *Theobroma* (blue) is performed by the MEGA Program. Green, blue, and red groups indicate TS, CS, and TcS (theacrine synthase gene) classes.

For the particular differences in the TCS1h amino acid sequence, further analysis of enzyme activity was required. In the presence of methyl donor *S*-adenosyl-_L_-methionine (SAM), the recombinant enzyme preparation was incubated with the substrate 7-methylxanthine or theobromine. HPLC detection revealed that TCS1h had similar functions to TCS1a, TCS1d, TCS1e, and TCS1f, and it had both theobromine synthase (TS) and CS activities. The CS/TS activity ratio (56.3%) was higher than that of TCS1a **(**29.3%, Fig. 2A; Fig. S2, see online supplementary material).

Phylogenetic tree analysis was performed using cDNA sequences of NMTs related to caffeine synthesis in *Camellia*, *Coffea*, and *Theobroma***(**Fig. 2B**)**. Combining the above differences in enzyme activities, the result showed that NMTs of different species clustered independently, and the different allelic variations of *TCS1* and *TcS* (theacrine synthase gene) [33] in tea plants could be clustered into three discriminative branches: TS, CS, and TcS classes. *TCS1b*, *TCS1c*, *TCS1g*, and *TCS1i* were clustered in the TS class, and their expressed proteins only had TS activity. *TCS1h* was clustered with *TCS1a*, *TCS1d*, *TCS1e*, and *TCS1f* into the CS class, and the expressed proteins of this class had both TS and CS activities.

To explore key sites affecting enzyme activity of TCS1, we performed site-directed mutagenesis on amino acids at the 153th [34], 221th or 225th, and 269th positions. These were the residues affecting the TCS1 activity [21] of the proteins encoded by three *TCS1* alleles (*TCS1a*, *TCS1c*, and *TCS1h*) **(**Fig. 2A**)**. The result showed that the activity of TS decreased significantly, and the activity of CS disappeared after the mutation of Arg225His in TCS1a. The activities of TS and CS decreased to less than 0.5 pkat mg^−1^ protein through the mutation of the 269th residue from serine (Ser) to proline (Pro) (this mutation did not exist in tea plants). When the 221th residue of TCS1c was mutated from His to Arg, it still did not restore CS activity, and it only had TS activity, indicating that other sites also affected the CS activity of TCS1c. When the 153th residue of TCS1h was mutated from tyrosine (Tyr) to His, the activities of TS and CS decreased significantly, and the ratio of the CS/TS activity was decreased (29.0%). These results indicated that the 225th and 269th amino acids are the key residues that determined whether or not TCS1 has CS activity, and the 153th amino acid significantly affected the TCS1 activity.

### Analysis of purine alkaloids content and *TCS1* expression in tea germplasms

**Figure 3:**
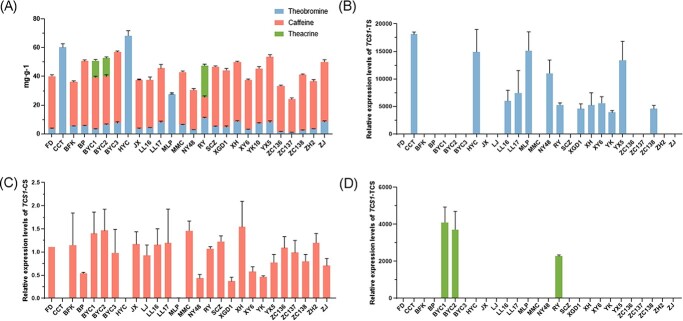
Expression level analysis of *TCS1* and *TcS* in 27 accession tea plants with diverse genetic background. **A** Purine alkaloid contents in 27 accessions. **B**–**D** Determination of *TCS1* allele expression levels in 27 accessions of tea plants by qRT-PCR. Primer pairs TCS1-TS, TCS1-CS, and TcS were used to detect gene whose encoded protein had only theobromine synthase (TS), both TS and caffeine synthase (CS), and TcS (theacrine synthase) activities, respectively.

To verify the correlation between the above three different discriminative branches and the content of purine alkaloids in tea plants, we determined purine alkaloids of 27 accessions of tea germplasms with diverse genetic backgrounds by ultra-high performance liquid chromatography (UPLC, Fig. 3A**)** before clarifying the respective expression level of alleles with distinct functions, and the gene expression level was detected by quantitative real-time PCR (qRT-PCR, Figs 3B, C, and D**)**. These results show that in ‘Malipo’ (MLP) (a wild tea plant from Yunnan, China), CCT, and HYC containing high or only theobromine, the expression level of *TCS1* alleles of the CS class was extremely low or not detected, which also proved the specificity of the primers. The tea plants contained theobromine and caffeine, when *TCS1* alleles of the CS class were expressed with high levels. In BYC1, BYC2, and RY containing theacrine, the expression level of *TcS* was high, whereas those that do not contain theacrine were extremely low. Therefore, we conceived that the contents of purine alkaloids in tea plants were related to the expression of corresponding functional genes and alleles, and the absence or presence and level of gene expression determined the content of purine alkaloids in tea plants to a certain extent.

### Activity analysis of *TCS1* promoters and identification of the key *cis*-acting element

**Figure 4:**
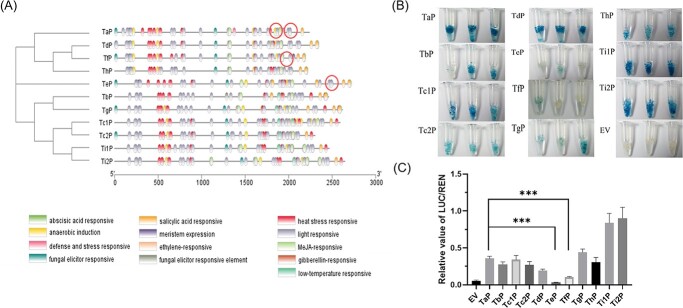
*Cis*-acting elements and promoter activity analysis of different *TCS1* alleles. **A** Distribution of *cis*-acting elements across the promoter sequences of different *TCS1* alleles. Predictive analysis with online software PlantCARE. **B** GUS histochemical staining of T_3_ generation *Arabidopsis thaliana* transduced with different promoters of *TCS1* alleles. Red circles indicate important variations in the light-response element. **C** Determination of the promoter activity of different *TCS1* alleles by dual-luciferase assay (*n* = 6); the difference in promoter activity was determined by detecting the ratio of firefly luciferase and renilla luciferase activities with an empty vector as the control; ***P < 0.001. EV, empty vector.

The sequences of different *TCS1* promoters are fairly variable (Fig. 1A), which may lead to significant differences in transcription levels. To investigate the mechanism of gene differential expressions, we cloned the promoters of different alleles including *TCS1h* from specific tea germplasms **(**Tables S1 and S5, see online supplementary material). Among them, two different *TCS1* promoter sequences were amplified from tea plants containing *TCS1c* and *TCS1i* alleles. The 5′ end sequences of the 11 different *TCS1* alleles (sequences before the start codon ATG) were defined as the responding promoters, namely, TaP, TbP, Tc1P, Tc2P, TdP, TeP, TfP, TgP, ThP, Ti1P, and Ti2P. Although amplified with the same primers, they varied widely in sequences from 2193 to 2715 bp. The variation of their similarity ranged from 78.6% to 98.8% (Table S6, see online supplementary material). It showed the number of different functional *cis*-acting elements (except for the TATA box and CAAT box) and the distribution on the promoter sequence of different *TCS1* alleles (Fig. 4A; Fig. S3, see online supplementary material). Each promoter had elements related to light response. TbP, Tc1P, Tc2P, TgP, Ti1P, and Ti2P had *cis*-acting elements with low-temperature response, whereas TaP, TdP, TeP, TfP, and ThP did not have it. In addition, a segment of the Tc1P sequence was found at the 3′ end with a large number of elements, whereas Tc2P with high similarity did not have this feature.

To verify promoter activities, we recombined promoters of different *TCS1* alleles (greater than 2100 bp) into the PBI1101.3-GUS plus vector and transformed into *A. thaliana* (Fig. 4B). Based on the histochemical staining results of T_3_ transgenic *A. thaliana*, the promoter activities of *TCS1*e and *TCS1f* were significantly lower than the remaining promoters. Dual-luciferase assays (Fig. 4C) also demonstrated that *TCS1e* and *TCS1f* promoters had low activities. The results also suggest that molecular mechanism of low caffeine in MLP may be for the low transcription level of *TCS1f*, though *TCS1f* encoding protein with CS activity.

**Figure 5:**
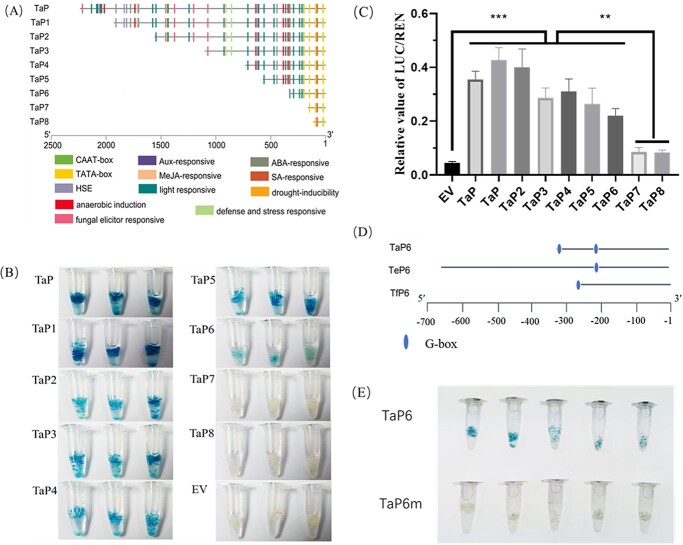
Promoter activity analysis of the key regions and *cis*-elements of truncate promoters of *TCS1a*. **A** Distribution of *cis*-acting elements on the *TCS1a* promoter sequence with deletions of different lengths. **B** GUS histochemical staining of T_3_ generation of *Arabidopsis thaliana* transfected with 5′ end fragment deletion of *TCS1a* promoter. **C** Determination of the promoter activity of different deleted fragments of the *TCS1a* promoter (*n* ≥ 6); ***P < 0.001, **P < 0.01. **D** Schematic representation of large indel mutations in *TCS1e* and *TCS1f* and the absence of a G-Box *cis*-element. **E** Comparison of GUS histochemical staining between TaP6 and mutant promoter. TaP6 represents the 331 bp of the *TCS1a* promoter, TeP6 and TfP6 represent the corresponding promoter sequences of *TCS1e* and *TCS1f*, and TaP6m represents a G-Box element near the 3′ end mutated with one base (G-214A). EV indicates empty vector.

To further figure out the internal mechanism of different caffeine content in tea plants, based on the ubiquitous *TCS1* allele in tea plants, we deleted the *TCS1a* promoter (TaP) at the 5′ end. As shown in Fig. 5A, the deletion of the TaP 5′ end fragment results in a gradual decrease in the number and types of elements. GUS histochemical analysis and dual-luciferase assay conferred that a segment between −331 and −178 was the basic region for starting *TCS1*a transcription **(**Fig. 5B and C**)**. Due to the low activities of promoters, we further compared the promoter sequences of *TCS1e* and *TCS1f* with other *TCS1* alleles and found that large fragments of insertion or deletion mutations occurred in them, and a G-box (CACGTG) *cis*-acting element was missing **(**Fig. 5D**)**. Through the experiments of site-directed mutagenesis, we found that the activity almost disappeared, when a G-box *cis*-acting element (near the 3′ end) in the 331 bp *TCS1a* promoter sequence was mutated **(**Fig. 5E**)**. In summary, we believed that the key *cis*-element in the promoter had a certain influence on the activity of different promoters, thereby affecting the caffeine content in different tea plants.

## Discussion

Some progress had been made in the alleles of *TCS1*, but it had abundant allelic variation due to the independent and recent rapid evolution mechanism of *NMTs* in tea plants [29]. Through the deeply functional identification of *TCS1* alleles, it showed that the diversity of *TCS1* sequence leads to the variations of *TCS1* enzyme activity or transcription level, affecting the different distribution patterns of purine alkaloids in tea plants. Previous studies indicated that the mutation of His at the 225th position to Arg in TCS1 would alter the steric hindrance of enzyme binding to substrates [21]. Therefore, the 225th amino acid residue was Arg or His, determining whether or not TCS1 had CS activity. In addition, this study found that the 221th amino acid residue (corresponding to the 225th amino acid residue of TCS1a) of TCS1c with only TS activity was mutated from His to Arg through site-directed mutagenesis, and it indicated that more than one sites affected the CS activity of TCS1c. The important role of the 269th amino acid residue in TCS1 in activity and substrate recognition was demonstrated by the mutation of Ser269Cys [21], but no mutation with a ring structure (such as Pro) was observed at 269th amino acid in tea plants. Through an interesting artificial mutation (Ser269Pro), the nearly disappeared enzymatic activity of TCS1 indicated that the 269th amino acid of TCS1 was the key active site determined by the CS activity.

Tea plants containing *TCS1e* or *TCS1h* also contain at least one other allele with the same function. Therefore, it is not feasible to analyse the expression levels of *TCS1e* or *TCS1h* by qRT-PCR. Thus, we investigated the difference in promoter activity of different *TCS1* alleles to analyse the internal mechanism of the difference in expression levels of different alleles. The low activities of the promoters *TCS1e* and *TCS1f* were confirmed by transient and stable transformation. Deletion of the 5′ end fragment essentially identified a basic activity region (between −331 and −178). Sequence alignment revealed that *TCS1e* and *TCS1f* had a large InDel mutation, and one G-Box *cis*-acting element was missing. The activity of the 331 bp *TCS1a* promoter was nearly abolished during a single-base mutation in the G-box *cis*-acting element (near the 3′ end). In conclusion, G-Box was a *cis*-acting element that critically affects the *TCS1* promoter activity, which may also lead to significantly lower activities of *TCS1e* and *TCS1f* than other alleles. This finding provided an important basis for the follow-up transcriptional regulation studies of *TCS1*.

Based on cluster analysis and the above-mentioned results, nine *TCS1* alleles are classified into three types: type I, the coding proteins only have TS activity and high promoter activities (*TCS1b*, *TCS1c*, *TCS1g*, and *TCS1i*); type II, the coding proteins have both TS and CS activities, but low promoter activities (*TCS1e* and *TCS1f*); and type III, the coding proteins have TS and CS activity, and the activities of promoter are high (*TCS1a*, *TCS1d*, and *TCS1h*). When tea plants contained only *TCS1* alleles of types I and II, they are low in caffeine, such as CCT (*TCS1c*), HYC (*TCS1i*), and MLP (*TCS1b* and *TCS1f*). Although these alleles with the ability to innovate low-caffeine tea germplasms were proposed in 2016 [21], previous cross-breeding practices had shown that low-caffeine breeding could not be achieved by aggregating *TS* alleles (*TCS1b*, *TCS1c*, *TCS1g*, and *TCS1i*). For example, no low-caffeine individual appeared in the offspring of ‘Hualing 7’ × CCT-F_1_**(**Fig. S4, see online supplementary material**)** and another three similar hybrid combination populations. Therefore, low-caffeine breeding aims to ensure that the offspring of the hybrid tea plants do not contain the *CS* allele with a high transcript level. In wild tea plants without caffeine, such as HYC and CCT, only TS alleles were contained. Theobromine and caffeine contents of the offspring HYC × CCT-F_1_ (the F_1_ individuals of CCT opening pollination) showed that this hybrid method could obtain low-caffeine offspring (Fig. S5, see online supplementary material). However, F_1_ offsprings of CCT, HYC, and MLP have poor adaptability, poor growth potential, and low survival rate. Using the specific genetic resource RY (containing one low transcription level allele *TCS1e* with CS activity) as the parent of low-caffeine breeding, it shows strong adaptability and similar growth potential to cultivated tea plants, and is more conducive to the rapid breeding of low-caffeine cultivars. Therefore, the development and utilization of *TCS1e* were more important.

**Figure 6:**
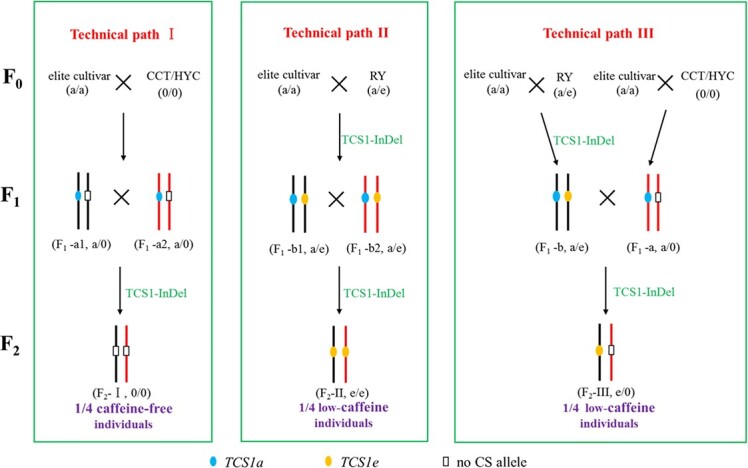
The strategy for enhancing high-theobromine and low-caffeine tea germplasms.

According to the above-mentioned results and breeding practice of the past 10 years, combined with the growth potential and adaptability of the F_1_ generation, we proposed three high-efficiency breeding strategies with high theobromine and low caffeine **(**Fig. 6**)**. Path I is the use of wild-specific resources, such as HYC and CCT, which contain only the TS alleles to cross with elite cultivars and obtain F_1_ offspring with better growth potential. Then, caffeine-free tea plants will be obtained by crossing between F_1_ offspring. Path II is aggregating *TCS1e* in RY with lower transcript levels. F_1_ offspring with better growth potential is obtained by crossing RY with elite cultivar. Path III is the mutual crossing of the two above-mentioned types of F_1_ offspring, innovating different high-theobromine and low-caffeine tea plants. The TCS1-Indel molecular marker can be used to rapidly screen different *TCS1* genotypes of hybrid offspring and assist in the selection of target individuals. Wild tea plants rich in theacrine can also be used to innovate new germplasms with high theacrine and low caffeine [33].

## Materials and methods

### Plant materials

A total of 673 tea accessions (Table S7, see online supplementary material) were used for screening rare *TCS1* allelic variations, including 200 accessions (primarily *C. sinensis* (L.) O. Kuntze and *C. sinensis var. assamica* (Masters) Kitamura) collected from the China National Germplasm Hangzhou Tea Repository (Hangzhou, China) in Zhejiang province and 473 accessions (primarily *C. taliensis* (W.W. Smith) Melchior, *C. sinensis var. assamica* (Masters) Kitamura, and the intermediate types of them) collected from Lincang city, Yunnan province, China, were used as experimental materials. A total of 27 typical tea accessions with clear genetic background information, as described in our previous study [35], were used for purine alkaloid determination and specific allele expression analysis. They were collected from their native habitat and are currently maintained in the China National Germplasm Hangzhou Tea Repository (Hangzhou, China).

### Detection of purine alkaloids and UPLC conditions

The biochemical samples were ground by using a micropipette grinder (Verder Shanghai Instruments and Equipment Co., Ltd, Shanghai, China) and assayed using UPLC. The content of caffeine, theobromine, and theacrine in the samples was determined, and each sample was independently repeated three times. The pretreatment of the sample and the UPLC measurement conditions were referred to the determination method in our previous study [36].

### Screening for novel alleles by using the TCS1-InDel marker

Based on the previous study [21], we designed specific primer pairs for the identification of *TCS1* allelic variation: TCS1PInDel-F and TCS1PInDel-R **(**Table S2, see online supplementary material). PCR amplification was performed using KOD-Plus-Neo (Toyobo, Co., Ltd, Osaka, Japan) with a 25 μL reaction system under the following conditions: 94°C for 2 min; 30 cycles of 94°C denaturation for 10 s, 51°C annealing for 25 s, 72°C extension for 45 s, extension at 72°C for 5 min.

### Sequence analysis

Sequence assembly and alignment used DNAMAN (v9.0) and ClustalW with BoxShade in EXPASY to output homologous alignment results. A phylogenetic tree was constructed in MEGA (v7.0) using the Neighbor-joining method and 1000 repeat bootstrap. The gene structure was visualized using Gene Structure Display Server (v2.0). Gene sequence information was visualized using TBtools.

### Gene expression analysis by qRT-PCR

Total RNA was extracted using the plant RNA kit (Aidlab Biotechnologies Co., Ltd, Beijing, China) following the manufacturer’s recommendations. The purity and concentration of RNA was certified using agarose gel electrophoresis and a spectrophotometer (Implen, CA, USA). First-strand cDNA was synthesized using the FastKing cDNA First-Strand synthesis kit (Tiangen Biotech Co., Ltd, Beijing, China) according to the manufacturer’s protocols **(**Table S3, see online supplementary material**)**. *Cs18S* was used as the reference gene. Table S2 (see online supplementary material) lists the specific primer pairs for qRT-PCR of TCS1-CS, TCS1-TS, and TcS designed according to 3′ end difference. Real-time PCR was similar to that presented in our recent study [33].

### Protein purification and activity assay of recombinant *TCS1* alleles

The full-length coding region of *TCS1h* was inserted into the pMAL-c5x vector (NEB, Beijing, China). The recombinant expression vector was introduced into *BL21* (DE3) *pLys* cells (TransGen Biotechnologies Co., Beijing, China). The target protein was purified by using the fusion protein label (MBP) on the expression vector, and an empty load was used as the negative control. The fusion expression of the target gene in *Escherichia coli*, the detection of the target protein, and the quantification of the purified protein were investigated as described by Jin *et al.* [21]. Each protein was determined in triplicate, and the activity was determined by HPLC.

### Promoter activity assay of *TCS1*

The promoter activity assay consists of the stable *A. thaliana* transformation and tobacco dual-luciferase reporter assay. The *TCS1* promoter sequence was recombined into PBI1101.3-GUS plus vector to construct a *GUS* expression vector. The successfully ligated binary expression plasmids were transferred into EHA105 competent cells (BioMed, BC303–01) using the freeze–thaw method. T_3_ generation of transgenic positive plants was obtained after *A. thaliana* inflorescence infection, and then GUS histochemical staining was performed. These GUS staining experiments were conducted on the basis of the instructions of the GUS staining kit (Gcloning Co., Ltd, Beijing, China). The *TCS1* promoter sequence was recombined into the pGreenII0800-Luc vector, and then the plasmids verified by sequencing were transformed into GV3101 (pSoup-19). The dual-luciferase activity assay was in accordance with our recent study [33].

### Statistical analyses

Data are presented as mean ± standard deviation (SD) for the relative expression of *TCS1* and relative value of the ratio of firefly luciferase and Renilla luciferase activities. Statistical significance of differences among groups was determined by Student’s *t*-test using SPSS (Chicago, IL, USA). Graphing was performed using GraphPad Prism 8.

## Supplementary Material

Web_Material_uhac279

## Data Availability

All data supporting the findings of this study are available within the paper and within its supplementary materials published online.
