# Supplementary material for: Deeply functional identification of TCS1 alleles provides efficient technical paths for low-caffeine breeding of tea plants
Source: Hortic Res. 2022 Dec 21;10(2):uhac279. doi: 10.1093/hr/uhac279 (PMC9926157; doi:10.1093/hr/uhac279)
Supplement: Web_Material_uhac279 [file web_material_uhac279.zip › Figure S1-5.docx]

**
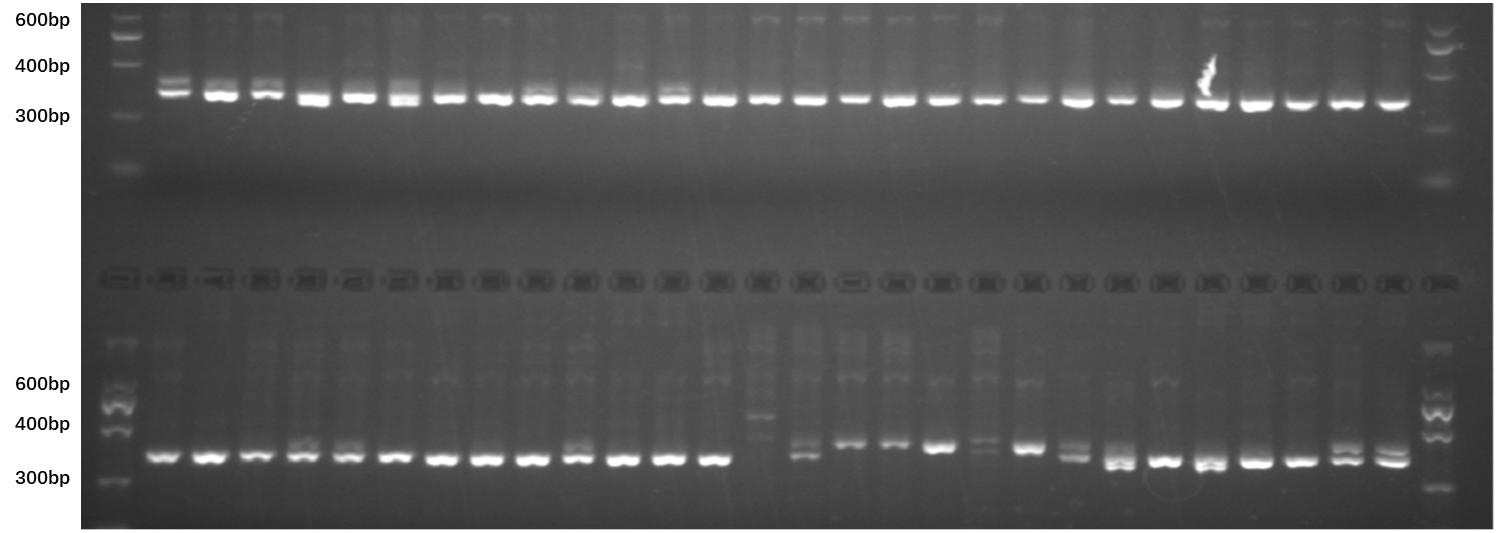
**

Figure S1. Screening of novel *TCS1* alleles in tea plants using the TCS1-InDel marker.


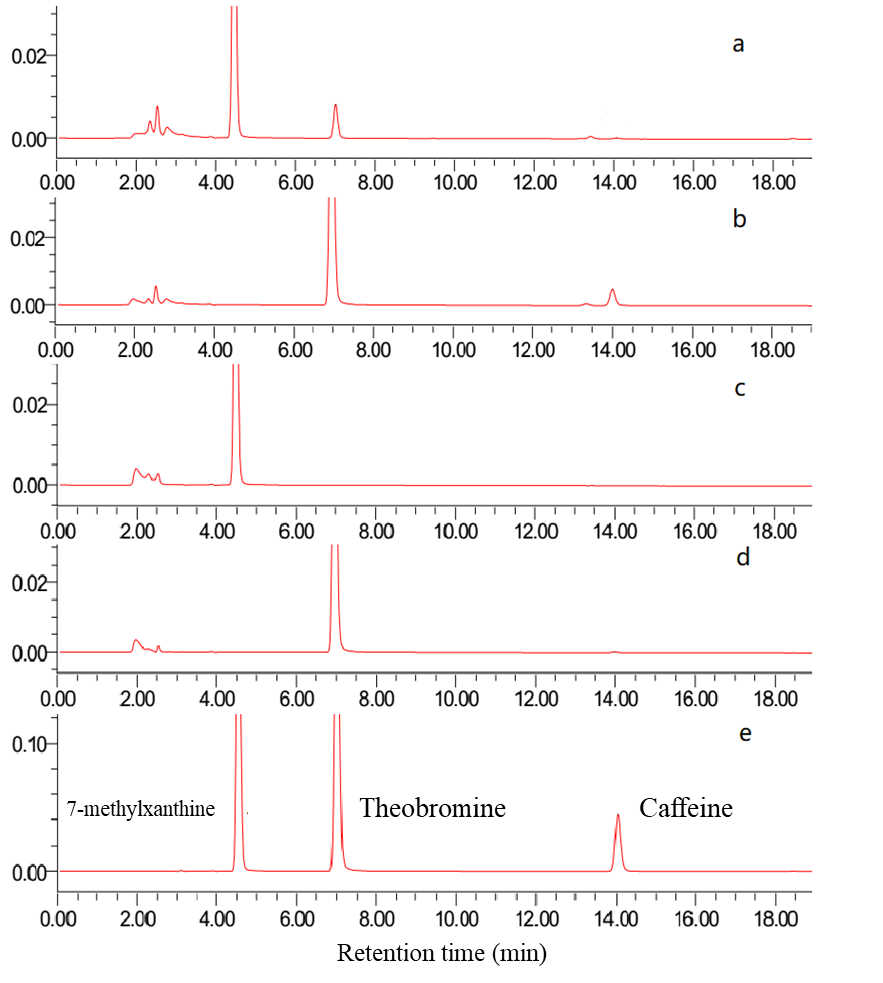


Figure S2. *In vitro* functional analysis of *TCS1i* by HPLC. a (substrate was 7-methylxanthosine) and b (substrate was theobromine), products of enzymatic assays with recombinant protein pMAL-TCS1h; c (substrate is 7-methylxanthosine) and d (substrate is theobromine), products of enzymatic assays with pMAL-c5x; e, standard compounds.

**

**

Figure S3. Heat map of *cis*-acting elements of promoters in different *TCS1* alleles. The right, left, top, and bottom were labeled as the *cis*-element types, *cis*-element functions, total number of *cis*-elements, and evolution of the promoter sequences, respectively.

**

**

Figure S4. Breeding practice for innovating low-caffeine tea plants by ‘Hualing 7’ × ‘CCT-F_1_’. ‘Hualing 7’ is the female parent, and the natural hybrid F_1_ offspring of ‘CCT’ is the male parent.

**
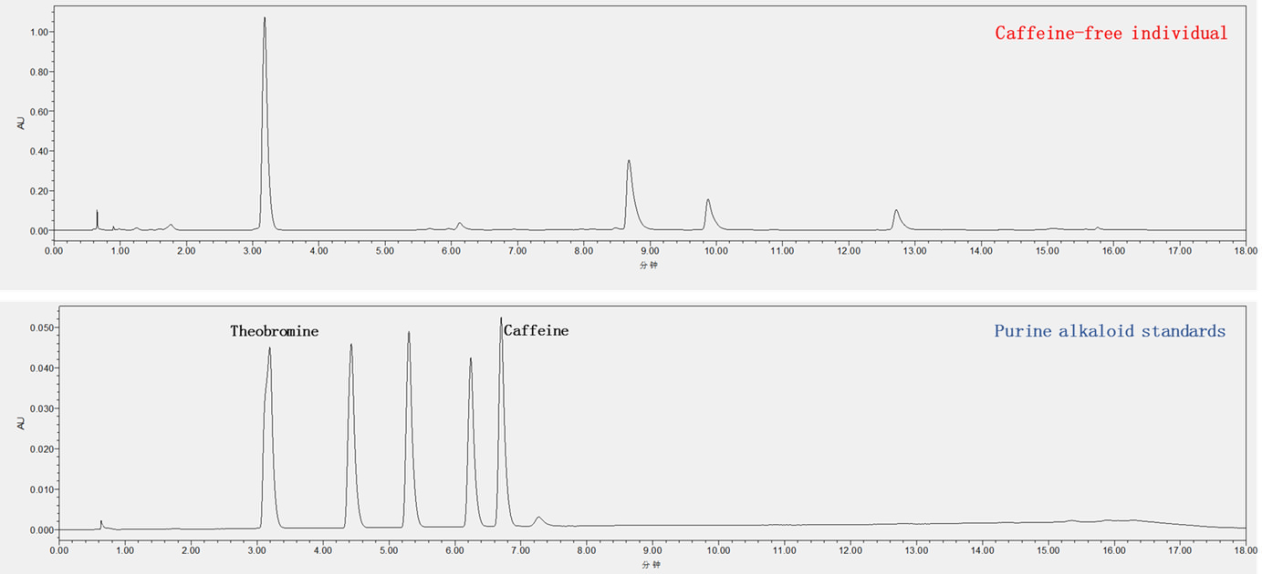
**

Figure S5. Determination of purine alkaloid contents in F_1_ individuals of ‘HYC’ × ‘CCT-F_1_’ by UPLC.
