# Supplementary material for: Deeply functional identification of TCS1 alleles provides efficient technical paths for low-caffeine breeding of tea plants
Source: Hortic Res. 2022 Dec 21;10(2):uhac279. doi: 10.1093/hr/uhac279 (PMC9926157; doi:10.1093/hr/uhac279)
Supplement: Web_Material_uhac279 [file web_material_uhac279.zip › Table S1-6.docx]

**Table S1.** *TCS1* alleles and purine alkaloid contents in different tea plants (mg g^-1^).

| Germplasm | Scientific name | *TCS1* alleles | Theobromine | Caffeine | Theacrine |
| --- | --- | --- | --- | --- | --- |
| FD | *Camellia sinensis* var. *sinensis* | a | 3.68 ± 0.34 | 36.51 ± 1.05 | ND |
| MLP | *C. gymnogyna* | b, f | 27.65 ± 1.08 | 0.12 ± 0.05 | ND |
| CCT | *C.* sp. | c | 60.27 ± 2.45 | ND | ND |
| NY48 | *C. gymnogyna* | b, d | 2.96 ± 0.13 | 27.52 ± 1.07 | ND |
| RY | *C.* sp. | a, b, e | 11.06 ± 0.56 | 14.52 ± 0.91 | 21.87 ± 1.12 |
| LL17 | *C. taliensis* | a, g | 7.95 ± 0.94 | 37.73 ± 2.67 | ND |
| ZJ | *C. sinensis* var. *assamica* | a, h | 8.29 ± 0.82 | 41.85 ± 1.50 | ND |
| HYC | *C.* sp. | i | 68.35 ± 3.41 | ND | ND |

ND: not detected.

**Table S2.** Primer sequences were used in the study.

| Primer name | Primer sequence（5ʹ-3ʹ） | Function |
| --- | --- | --- |
| TCS1P InDel F | TATGTCATGTTTCTATTATTT | Screening for *TCS1* alleles |
| TCS1P InDel R | TACTTTCTCCTTCTCCTCTGT |  |
| TCS1cDNAF | CACTGCTGTGGCAGCTGGC | Amplification of cDNA sequences |
| TCS1cDNAR | CAACTTCTCATTTCTCCCAAC |  |
| 18S-F | TCTCAACCATAAACGATGCCGACCAG | Expression quantification of reference genes |
| 18S-R | TTTCAGCCTTGCGACCATACTCCC |  |
| TCS1-TS-F | TAAAAATTACTTTTCT/TCCGACGAGGCG | Detection of gene expression levels |
| TCS1-TS-R | GTCCGCTGCAKTAAGAGCTKGAAGAA |  |
| TCS1-CS-F | TAAAARTTACTTTTCYGACGAGGCA |  |
| TCS1-CS-R | GTCYGCTGCGTTAAGAGCTTGAAGG |  |
| TcS-F | TAAAAATTACTTTTCTGACGAGGCA |  |
| TcS-R | CYGCTGCGTTAATAGCTTGAAAAAG |  |
| TaPF | ACGCGTCGACGTAGGTCAAGAATGTGGGTTTA | Amplifying the promoter sequence of *TCS1a* |
| TaPR | CGGGGTACCGCCTCGTCGGAAAAGTAAT |  |
| TaPF1 | ACGCGTCGACAAAATACGAAAAGTGTACTGA | 5ʹ deletion of the promoter sequence *TCS1a* |
| TaPR | CGGGGTACCGCCTCGTCGGAAAAGTAAT |  |
| TaPF2 | ACGCGTCGACCTGCTCGCCCTAGCATGATT |  |
| TaPR | CGGGGTACCGCCTCGTCGGAAAAGTAAT |  |
| TaPF3 | ACGCGTCGACAACTTTATCTCAATTTGGTC |  |
| TaPR | CGGGGTACCGCCTCGTCGGAAAAGTAAT |  |
| TaPF4 | ACGCGTCGACGGCAAGTTCGAGATTGTACTA |  |
| TaPR | CGGGGTACCGCCTCGTCGGAAAAGTAAT |  |
| TaPF5 | ACGCGTCGACTCTTTCTTATCTTTTCCTCTCAA |  |
| TaPR | CGGGGTACCGCCTCGTCGGAAAAGTAAT |  |
| TaPF6 | ACGCGTCGACTCACGTGGCGTACTACTTACC |  |
| TaPR | CGGGGTACCGCCTCGTCGGAAAAGTAAT |  |
| TaPF7 | ACGCGTCGACTAACTTAGGCGTACCCGAGC |  |
| TaPR | CGGGGTACCGCCTCGTCGGAAAAGTAAT |  |
| TaPF8 | ACGCGTCGACCTTCAGGCCATTATTCACAT |  |
| TaPR | CGGGGTACCGCCTCGTCGGAAAAGTAAT |  |
| TaPF | ACGCGTCGACGTAGGTCAAGAATGTGGGTTTA | Amplification of the promoter sequence of the *TCS1* alleles |
| TbcgiPR | CGGGGTACCAGTAGCTAGCTCCACGCCT |  |
| Tbc1i1i2PF | ACGCGTCGACGTAGGTCAGAATGTGGGTTTA |  |
| TbcgiPR | CGGGGTACCAGTAGCTAGCTCCACGCCT |  |
| TgPF | ACGCGTCGACGTAGGTCAAGAATGTGGGGTTTA |  |
| TbcgiPR | CGGGGTACCAGTAGCTAGCTCCACGCCT |  |
| TaPF | ACGCGTCGACGTAGGTCAAGAATGTGGGTTTA |  |
| TdfhPR | CGGGGTACCGCCTCGTCAGAAAAGTAAT |  |
| ThPF | ACGCGTCGACGTAGTCAAGAATGTGGGTTTA |  |
| TdfhPR | CGGGGTACCGCCTCGTCAGAAAAGTAAT |  |
| TePF | CCCAAGCTTGTAGGTCAAGAATGTGGGTTTA |  |
| TePR | CGGGGTACCGCCTCGTCAGAAAAGTAAC |  |

**Table S3.** Coding region nucleotide sequence of different *TCS1* alleles.

| >TCS1g  ATGGGGAAGGTGAACGAAGTGTTGTTCATGAACAGAGGAGAAGGAGAAATTAGTTATGCACAAAACTCTTCTTTCACACAAAAAGTGGCCTCAATGGCAACGCCAGCGTTAGAAAATGCAGTTGAAACTCTCTTCTCCAAAGATTTCCACCTTCTTCAAGCTCTTAATGCAGCGGACTTGGGTTGTGCAGCGGGTCCAAACACGTTCGCAGTGATTTCTACGATCAAGAGAATGATGGAAAAGAAATGCAGGGAATTGAATTGCCAAACACTGGAACTTCAGGTTTACTTGAATGATCTTTTTGGAAACGATTTCAATACCCTCTTCAAAGGCCTGTCGTCTGAGGTTGTTGGTAACAAATGTGAGGAAGTTTCTTGTTATGTGATGGGAGTACCGGGGTCTTTCCATGGCCGGCTTTTTCCTCGTAACAGCTTGCATTTAGTTCATTCCTCTTACAGTGTTCATTGGCTTACTCAG  GAACCAAAAGGACTCACAAGCAGAGAAGGCTTGGCATTAAACAAGGGGAAGATTTACATATCAAAGACAAGCCCTCCTGTTGTAAAAGAAGCCTACTTATCTCAATTTCATGAAGATTTCACAATGTTTCTCAACGCTAGATCCCAAGAGGTGGTTCCAAATGGTTGTATGGTGTTGATACTTCATGGTAGGCAATCTTCTGATCCTTCAGAGATGGAGAGCTGCTTTACTTGGGAACTATTAGCTATAGCCATTGCTGAATTGGTTTCACAG  GGATTGATAGATGAAGATAAATTAGACACCTTCAATGTACCTAGCTATTTTCCATCACTTGAGGAAGTGAAAGACATAGTGGAGAGGGACGGATCATTCACAATTGATCATTTGGAGGGGTTTGAACTTGATAGCCTAGAGATGCAAGAGAATGATAAATGGGTTAGAGGGGACAAGTTTGCCAAGATGGTCAGGGCCTTCACAGAGCCTATAATTTCAAACCAGTTTGGACATGAAATCATGGACAAACTATATGACAAATTCACTCACATTGTAGTTTCAGATTTGGAAGCAGAGCTACCGAAGACCACAAGTATCATCCTAGTGCTTTCCAAGATTGTTGGATAGGATTGATGGATAG |
| --- |
| >TCS1h  ATGGAGATAGCTACTACGGGGAAGGTGAACGATGTGTTGTTCATGAACAGAGGAGAAGGAGAAAGTAGTTATGCACAAAACTCTTCTTTCACGCAACAAGTGGCCTCAATGGCACAGCCAGCGCTAGAAAATGCAGTTGAAACTCTCTTCTCCAAAGATTTCCACCTTCAAGCTCTTAACGCAGCGGACTTGGGTTGTGCAGCGGGTCCAAACACATTCGCAGTGATTTCTACGATCAAGAGAATGATGGAAAAGAAATGCAGGGAATTGAATTGCCAAACACTGGAACTTCAGGTTTACTTGAATGATCTTTTTGGAAATGATTTCAATACCCTCTTCAAAGGCCTGTCGTCTGAGGTTATTGGTAACAAATGTGAGGAAGTTCCGTGTTATGTGATGGGAGTACCGGGGTCTTTCCATGGCCGGCTTTTTCCTCGTAACAGCTTACATTTAGTTTATTCCTCTTACAGTGTTCATTGGCTTACTCAGGCACCAAAAGGACTCACAAGCAGAGAAGGCTTGGCATTAAACAAGGGGAAGATTTACATATCAAAGACAAGCCCTCCTGTTGTAAGAGAAGCCTACTTATCTCAATTTCATGAAGATTTCACAATGTTTCTCAATTATAGATCCCAAGAGATGGTTCCAAATGGTTGTATGGTGTTGATACTTCGTGGTAGGCAATGTTTTGATCCTTCAGACATGCAGAGCTGCTTTACTTGGGAACTATTAGCTTTAGCCATTGCTGAATTGGTTTCACAGGGATTGATAGATGAAGATAAATTAGACACCTTCAATATACCCAGCTATTTTGCATCACTTGAGGAAGTGAAAGATATAGTGGAGAGGGACGGATCATTCACAATTGATCATATAGAGGGGTTTGATCTTGATAGCGTAGAAATGCAGGAGAATGATAAATGGGTTAGAGGGGAAAAGTTTACCAAGGTTGTCAGGGCCTTCTCAGAGCCTATAATTTCAAGCCAGTTTGGACATGAAATCATGGACAAACTATATGACAAATTCACTCACATTGTAGTTTCAGATTTGGAAGCAAAGCTACCGAAGACCACAAGTATCATCCTAGTGCTTTCCAAGATTGATGGATAG |
| >TCS1i  ATGGGGAAGGTGAACGAAGTGTTGTTCATGAACAGAGGAGAAGGAGAAATTAGTTATGCACAAAACTCTGCTTTCACACAAAAAGTGGCCTCAATGGCAATGCCAGCGCTAGAAAATGCAGTTGAAACTCTCTTCTCCAAAGATTTCCACCTTCTTCAAGCTCTTACTGCAGCGGACTTGGGTTGTGCAGCGGGTCCAAACACGTTCGCAGTAATTTCTACGATCAAGAGAATGATGGAAAAGAAATGCAGGGAATTGTATTGCCAAACACTGGAACTTCAGGTTTACTTGAATGATCTTTTTGGAAACGATTTCAATACCCTCTTCAAAGGCCTGTCGTCTCAGGTTGTTGGTAACAAATGTGAGGAAGTTTCTTGTTATGTGATGGGAGTACCGGGGTCTTTCCATGGCCGGCTTTTTCCTCGTAACAGCTTGCATTTAGTTCATTCCTCTTACAGTGTTCATTGGCTTACTCAG  GCACCAAAAGGACTCACAAGCAGAGAAGGCTTGGCATTAAACAAGGGGAAGATTTACATATCAAAGACAAGCCCTCCTGTTGTAAAAGAAGCCTACTTATCTCAATTTCATGAAGATTTCACAATGTTTCTCAACGCTAGATCCCAAGAGGTGGTTCCAAATGGTTGTATGGTGTTGATACTTCATGGTAGGCAATCTTCTGATCCTTCAGAGATGGAGAGCTGCTTTACTTGGGAACTATTAGCTATAGCCATTGCTGAATTGGTTTCACAG  GGATTGATAGATGAAGATAAATTAGACACCTTCAATGTACCTAGCTATTGGCCATCACTTGAGGAAGTGAAAGACATAGTGGAGAGGGACGGATCATTCACAATTGATCGTTTGGAGGGGTTTGAACTTGATAGCCTAGAGATGCAAGAGAATGATAAATGGGTTAGAGGGGACAAGTTTGCCAAGATGGTCAGGGCCTTCACAGAGCCTATAATTTCAAACCAGTTTGGACATGAAATCATGGACAAACTATATGACAAATTCACTCACATTTTAGTTTCAGATTTGGAAGCAGAGCTACCGAAGACCACAAGTATCATCCTAGTGCTTTCCAAGATTGTTGGATAG |

**Table S4.** Coding region nucleotide (upper portion of matrix) and amino acid (bottom portion of matrix) sequence comparison (% similarity) between different *TCS1* alleles.

|  | TCS1a | TCS1b | TCS1c | TCS1d | TCS1e | TCS1f | TCS1g | TCS1h | TCS1i |
| --- | --- | --- | --- | --- | --- | --- | --- | --- | --- |
| TCS1a | - | 94.63 | 94.54 | 97.39 | 96.39 | 97.84 | 95.26 | 98.65 | 94.54 |
| TCS1b | 90.96 | - | 99.54 | 94.54 | 95.36 | 95.26 | 98.91 | 94.08 | 99.64 |
| TCS1c | 90.41 | 99.45 | - | 94.54 | 95.17 | 95.17 | 98.82 | 93.99 | 99.73 |
| TCS1d | 94.58 | 89.59 | 89.04 | - | 96.84 | 98.83 | 95.36 | 96.85 | 94.54 |
| TCS1e | 93.75 | 91.23 | 90.68 | 93.48 | - | 97.38 | 95.99 | 95.75 | 95.26 |
| TCS1f | 95.39 | 90.96 | 90.41 | 97.29 | 95.38 | - | 95.99 | 97.30 | 95.17 |
| TCS1g | 92.05 | 98.08 | 97.53 | 90.96 | 92.33 | 92.33 | - | 94.72 | 98.91 |
| TCS1h | 96.75 | 89.59 | 89.04 | 93.22 | 92.12 | 94.04 | 90.68 | - | 93.99 |
| TCS1i | 90.41 | 99.45 | 99.45 | 89.04 | 90.68 | 90.41 | 97.53 | 89.04 | - |

**Table S5.** Promoter sequences of different *TCS1* alleles.

| >TaP  GTAGGTCAAGAATGTGGGTTTATATGCATTATTTTGAAGGCGTTACAGTTTTGCTGTCATTTTTGGGCTGATTTGCTATTATTTTTGGGCTGATTTATCATATGGTTAAGCTTCATTTTGAACTCAAAAAAAAAAAAAAAAAAAAATTATGATAGGATAGATTAATAAGAAGAGATAGGTCAATCAAATTAATTCTTTCAAATAATCAAAAACATAATCAAAACCAAAACAAATTATCAAAATCAAATATGTCTAACAAAATACATAAGAATACGAAAAGTGTACTGAATTTTTGTATTTTTTGTATTTATATTTTTGGTAATTAGTAAATTTTTTAAAGATTTTTTTTACAAATATTATACTTTTTAAGTATATTTATGTTAGGTGTATCTAAAAATTTTAAAAAATTGCTTAGAATATTAAAAAAAATTTAGTGAAAACAAAAAAAAAAAATGAAAAAAAAAAATTCAATTTTCTAAAAAGTGAATCCTGAAAATTCAAACCAAACAAACTAAATTTGTTGTTAGAGTATTAAATAATTTCTCTATAAGTGTTTGTCAATTTTAGTACTTCTTTTAAAGTCTTAAGATTGATGTTTAAACTCATTAAATTGGATCAGAATTATTTTTTAAAGTTTTTCGTAGATGGGTCGGATTTGGAAATGTTTCTTCCTGCTCGCCCTAGCATGATTATTAATATATATTATGTACATGTAAAATTTTACTATAAATACTTCTTATTGTATGTATTCCTAACTTATATTATACTTTTAAGTTATACAAATTTTTTTTTTCAGTTATAATTAATTTTTTTTTGTAGTTGCGATCTTATTTTAGTTATGACTCATAATATCTCATGTATTATTTGGTTGACTTTTTTTTTTGTTTTTTTGTTTTGACTTTAAAGTATGAAATTATGTCATTTTTTTTTTTAGTTATAAGGTGCATACGATACATACCCTTTAAATTTTTCTTGTATGTACATATGTACATATTAATATCTGTATATATATAATTTACAAAATTTTACTTAGAATAAAAAGATATACTTAAAATGAAAAATGTATTTATTTAAGTTAATATTTAAGATGTAATGACTAAATATTAAGGTGTAATTTTAATTAGCTCGATTTTGATTTTAAATTTTTAACTTTATCTCAATTTGGTCAAATGGATTACAACTCTTAATATTTTTTAATAAAAAAATATGTATATAAGTGTCATGTGTCTCAAAATTATGAATTTATCTAACTCAGTAAGTGAACCTCAACTAATTTAGTGATAATTTTTCTTAAAAAACACTAATGATATTATTAATTTGATCAAATTGACGTAAAATCTAAAATTAAGGATCAAAATCAAATCAATTATAAATGTAAAAACTAAAATAAACAAAAGATAAAATATAAGGACATCCGTGTAATTCACCCACAAAATTATCATTTTTCAGTTTTATAATATTTTAAATTGTTTATATGAGTTTGTTGGGCAAGTTCGAGATTGTACTAGCAAGATTTTAACGCTAGCTTGGGAGGGATTTTGTGTTTGTTTGATTTGTATCTCATGATATAATTTTTAATTTTTATTTTTTTAAATTTTGTTTGGTTTAAATATTTTGAATTTTTTTTTTCAAAATCACTTTTTTTTCTGTATATATCAATCAGTCACTTTTTCTTTCTTATCTTTTCCTCTCAATCATTTTTTTTTCTCACACACATCTACTCAAACTACAATAAAATATCAAATCATCCCAAAATCTCAAATTATTTTCAAAATATACAACCAAACAAACTAAAAAAATTTCTAAACTATCTCTCAAAAAAAAAAAAATTAAAAATTTATCTCAAAACAAAAACCAAACACGCCCTTTTAGTTTCAAAAACTGAAAAAATATTTGGTTTTATTGGACGTCACGTGGCGTACTACTTACCAATAATAATATGTCATGTTTCTATTATTTTTTAATCACTTAATATAAAATTATAAATCTCATTTTTTTTATTAATTAAAATACTTGTGTATCACGTGCAAAATCAACCAATAATATCTCCAAAAAAAAATCCTAACTTAGGCGTACCCGAGCACCCAGACTATAGAGAGGCCTTCAGGCCATTATTCACATCACTGCTGTGGTAGCTGGCCTCTTTGCTATAAAAATTAGTGCTTTTCTGGTTATTCATATTCATATCACTGCTGTGGCAGCTGGCCTCTTTGCTATAAAAATTACTTTTCCGACGAGGC |
| --- |
| >TbP  GTAGGTCAGAATGTGGGTTTATATGCATTATTTTGAGGGCGTTACAGTTTTGCTATCGTTTTTGGGCTGATTTGCTGTTATTTTTGGGCTGATTTATCATATGTTTAAGCTTCATTTTGAACTCAAAAAAAAAACAAAAAAAAAACAAAACAAAACAAAATTAGAGCATCAAATAATCAATTAATCTAAGATGATTTAAGGAACTTATATCAGGCAAGATTGAATTTCATTTTGATCTTCAAAAATAACATTATGATAGGATAGATTAATATGAATAATTTGAGATATGTCAATCAAATTAATTCTTTCAAATAATCAAAAACATAATCAAAACAAAAACAAATTATCAAAATCAAATATGTCTAACAAAATACATAAGAATACGAAAAGTGTATTGAATTTTTGTATTTTTTGTATTTATATTTTTGGTAATTAGTGAATTTTTTAAAGATTTTTTTTACAAATATTATACTTTTTAAGTATATTTATGTTAGGTGTATCTAAAAATTTTAAAAAATTGCTTAGAATATTAAAAAAAATTTAGTGAAAAAAAAAATGTAAAAAAAAAAATTTCAATTTTCTAAAAATTGAATCCTGAAAATTCAAACCAAACAAACTAAATTTGTTGTTAGAGTATTAAATAATTTCTCTATAAGTGTTTGTCAATTTTAATACTTCTTTTAAAGTCTTAAGACTGATATTTAAACTCATTAAATTGGATCAGAATAATTTTTTAAAGTTTTTCGTAGATGGGTTGGATTTGAAAATGTTTCTTCCTGCTCGCCCTAGCATGATTATTAATATATATTATGTACATGTAAAATTTTACTATAAATACTTCTTATCGTATGCATTCTTAACTTATATTATACTTTTAAGTTATACAAATTTCTTTTTTAGTTATGATTAATTTTTTTTCTTTAGTTGCGATCTTATTTTAGTTATGACTCATAAGATCTTTGGTTATATCTCATGTATTATTTGGTTATGACTTATATATATATATATATATATATAGCTATGACTTTAAAGTATGAAGTTATGTTATTTTTCTTTTAGTTATAAGATGCATACGATACATACCTTTTAAATTTTTCTCGTATGTGCATATGTACATATTAATATCTGTATATATATAATTTACTAAATTTTATTTTTTTTAAATAAAAAGATATACTTAAAATGAAAAATGTATTTATTTAAGTTAATATTTAAGATGTAATGACTAAATATTAAGGTGTAATTTTAATTAGCTCGATTTTGATTTTAAATTTTTAACTTTGTCTCAATTTGGTCAAATGGATTACAACTCTTAATATTTTTTAATAAAAAATATATACATAAGTGTCATTTGTCTCAAAATTATGAATTTATCTAACTCAGTAAGTGAACCTCAACTAATTTAGTGATAATTTTTCTTAAAAAATACTAATGATATTATTAATTTGATCAAATTGACGTAAAATCTAAAATTAAGGATCAAAATCAAATCAATTAAAAATGTAAAAACCAAAATAAAATAAAAGATAAAATATAAGGACATCGGCGTAATTTACCCACAAAATTATCATTTTTCAGTTTTATGATATTTAAAATTGTTTATGTGAGTTTGTTGGGAAGTTCGAGATTGTACTAGCAAGATTTTAAGGCTAGCTTGGAGGGATTTTGATTTGTTTGATTTGTATCTCATGATATAATTTTTAATTTTTTTTTAAAATTTTGTTTGGTTTAAATTTTTTGAGTTTTTTTTTTTTCAAAATCAGTTTTTTTTTCGTATACATCAATCAGTCACTTTTTCTCTTTTCTCTTTTCTTCTCAATCATTTTTTTCTCTCACACATATCTACTCAAATTATAATAAAATATCAAATCATTCCAAACTCCCAAACTCCGAAACTATTTTCAAAATATACAACAAAACAAACCAAAAAAATTTCTAAACTCTGTTTCCAAAAAAAAAAATATTTAAAAATTTATCTCAAAACAAAAACCAAACACACCCTTTTAGTTTCAAAAACTGAAAAAAATATTTGGTGTTATTGGACGTCACGTGGCGTACTACTTACCAATAATAATATATCATGTTTCTATTATTTTTTAATCACTTAATATAAAATTATAAATCTTATTTTTTTTTATTAATTAAAATACTTTTGTGTATCACGTGACAATACCTCAAAATCAACCAATAATTTCTCTCCAAGTCGGGCCGGGTCAGATTTGGATTAAAGATTTTTCTTTTTTTTAAAAAAAAAATAAAATAAAAAAATTCCTAACTTAGGCGTAGCCGAACACCCAGACTATAGATAGGCTTTCAGGCCATTATTCACATCACTGCTGTGGCAGCTGGCCTCTTTGCTATAAAATGCTTTTCTGGTTATTCATATTCATATCACTGCTGTGGCAGCTGGCCTCTTTGCCATAAAAAATTACTTTTCTGACGAGGCGTGGAGCTAGCTACT |
| >Tc1P  GTAGGTCAGAATGTGGGTTTATATGCATTATTTTGAGGGCGTTACAGTTTTGCTGTCGTTTTTGGGCTGATTTGCTGTTATTTTTGGGCTGATTTATCATATGTTTAAGCTTCATTTTGAACTAAAAAAAAAAAAAAAAAAACAAAATTAGAGCATCAAATAATCAATTAATCTAAGATGATTTAAGGAACTTATATCAGGCAAGATTGAATTTCATTTTGATCTTCAAAAATAACATTATGATAGGATAGATTAATATGAATAATTTGAGATATGTCAATCAAATTAATTCTTTCAAATAATCAAAAACATAATCAAAACAAAAACAAATTATCAAAATCAAATATGTCTAACAAAATACATAAGAATACGAAAAGTGTACTGAATTTTCGTATTTTTTTTATTTATATTTTTGGTAATTAGTGAATTTTTTAAAGATTTTTTTTTACAAATATTATACTCTTTAAGTATATTTATGTTAGGTGTATCTAAAAATTTTTAAAAATTGCTTAGAATATTAAAAAAAATTTAGTGAACCCAAAAAAAAAAAAAAAAAAAAATTTTTTTTTTTTTTTTTTCAATTTTCTAAAAAGTGAATCCTAAAAATTCAAACCAAACAAACTAAATTTGTTGTTAGAGTATTAAATAATTTCTCTATAAGTGTTTGTCAATTTTAATACTTCTTTTAAAGTCTTAAGACTGATATTTAAACTCATTAAATTGGATCAGAATTATTTTTTAAAGTTTTTCTTGGATGGGTTGGATTTGGAAATGTTTCTTCTTGCTTGCCCTAGCATGATTATTAATATATATTATGTACATGTAAAATTTTACTATAAATACTTCTTATCGTAGGCATTCTTAACTTATATTATACTTTTAAGTTATACAAATTTTTTTTTCAGTTATGATTAATTTTTTTTCTTTAGTTGCGATCTTATTTTAGTTATGACACATAAGATCTTTGGTTATATCTCGTGTATTATTTGGTTATGACTTTATATATATATATATATAGCTATGACTTTAAAGAATGAAGTTATGTCATTTTTCTTTTAGTTATAAGGTGCATACGATACATACCCTTTAAATTTTTCTTGTATGTGCATATGTACATATTAATATCTGTATATATATAATTTACTAAATTTTACTTTTTTTAAATAAAAAGATATACTTAAAATGAAAAATGTATTTATTTAAGTTAATATTTAAGATGTAATGACTAAATATTAAGGTGTAATTTTAATTAGCTCGATTTTGATTTTAAAATTTTAACTTTGTCTCAATTTGGTCAAATGGATTACAACTCTTAATATTTTTTAATAAAAAATATGTACATAAGTGTCATGTGTCTCAAAATTATAAATTTATCTAACTCAGTAAGTGAACCTCAACTAATTTAGTGATAATTTTTCTTAAAAAATACTAATGATATTATTAATTTGATCAAATTGATGTAAAATCTAAAATTAAGGATCAAAATCAAATCAATTAAAAATGTAAAAACCAAAATAAAAAAAAAAATAAAATATAAGGACATCGGCGTAATTTACCCACAAAATTATCATTTTTCAGTTTTATGATATTTAAAATTGTTTATATGAGTTTGTTGGGCAAGTTCGAGATTGTACTAGCAAGATTTTAAGGATAGCTGGGAGGGATTTTGGATTTGTTTGATTTGTTTCTCATGATATAATTTAAAAATTTTTTTTTTAATTTTGTTTGGTTTAAATTTTTTGAGTTTTTTTTTTCAAAATCATTTTTTTTTCGTATACATCAATCAGTCACTTTTTCTCTTTTCTCTTTCCTTTTCAATAATTTTTTTTCTCTTACACATATCTACTCAAACTATAATAAAATATCAAATCATTCCAAACTCTCAAACTCCGAAACTATTTTCAAAATATACAACAAAATAAATCAAAAAAATTTCTAAACTCTCTCTCTAAAAAAAAAAAAAATATTTAAAAATTTATTTCAAAACAAAAACCAAACACACCCTTATAGTTTCAAAAACTGAAAAAATATTTGGGGTTCGTTTGGTTTGAATTTCGGAAAAATTTTTGGGAAATTTTCTCTACAAATAAGTTTTTAAAAGTAATGAAACCAAACAAATTTTCCCAAAAAAATTTGTCAAAACTTGTAAACCAAACAAGTTTTCCAAACAAATCTCTCTCTCACACACATCAAATCAATCACTTTTTCCAAAACTTTCTCTCAAAATAATTTTTCAAAACATCTTTTGAAAAAAACAAAACCAAACAATTTTTCATACTTTTTATCTCTAAAACACATCTCTCGAACACAAACCAAACATACCCTTGATGTTATTGGACGTCACGTGGTGTACTACTTACCAATAATAATATATCATGTTTCTATTATTTTTTAATCACGTGGCGTAGCCTAACATCCAGACTATAGATAGGCTTTCAGGCCATTATTCACATCACTACTGTGGTAGCTGGCCTCTTTACTATAAAAATTAGTGCTTTTCTGGTTATTCATATTCATATTCATATCACTGCTGTGGCAGCTGGCCTCTTTGCCATAAAAAATTACTTTCCGACGAGGCGTGGAGCTAGCTACT |
| >Tc2P  GTAGGTCAAGAATGTGGGTTTATATGCATTATTTTGAGGGCGTTACAGTTTTGCTGTCGTTTTTGGGCTGATTTGCTGTTATTTTTGGGCTGATTTATCATATGTTTAAGCTTCATTTTGAACTCAAAAAAAAAAAAAAAAAAAACAAAAAACAAAATTAGAGCATCAAATAATCAATTAATCTAAGATGATTTAAGGAACTTATATCAGGCAAGATTGAATTTCATTTTGATCTTCAAAAATAACATTATGATAGGATAGATTAATATGAATAATTTGAGATATGTCAATCAAATTAATTCTTTCAAATAATCAAAAACATAATCAAAACAAAAACAAATTATCAAAATCAAATATGTCTAACAAAATACATAAGAATACGAAAAGTGTACTGAATTTTCGTATTTTTTTTATTTATATTTTTGGTAATTAGTGAATTTTTTAAAGATTTTTTTTTACAAATATTATACTCTTTAAGTATATTTATGTTAGGTGTATCTAAAAATTTTTAAAAATTGCTTAGAATATTAAAAAAAATTTAGTGAACACAAAAAAAAAAAAAAAAAAAAATTTTTTTTTTTTTTTTTCAATTTTCTAAAAAGTGAATCCTAAAAATTCAAACCAAACAAACTAAATTTGTTGTTAGAGTATTAAATAATTTCTCTATAAGTGTTTGTCAATTTTAATACTTCTTTTAAAGTCTTAAGACTGATATTTAAACTCATTAAATTGGATCAGAATTATTTTTTAAAGTTTTTCTTGGATGGGTTGGATTTGGAAATGTTTCTTCTTGCTCGCCCTAGCATGATTATTAATATATATTATGTACATGTAAAATTTTACTATAAATACTTCTTATCGTAGGCATTCTTAACTTATATTATACTTTTAAGTTATACAAATTTTTTTTTCAGTTATGATTAATTTTTTTTCTTTAGTTGCGATCTTATTTTAGTTATGACTCATAAGATCTTTGGTTATATCTCGTGTATTATTTGGTTATGACTATATATATATATATATATATAGCTATGACTTTAAAGAATGAAGTTATGTCATTTTTCTTTTAGTTATAAGGTGCATACGATACATACCCTTTAAATTTTTCTTGTATGTGCATATGTACATATTAATATCTGTATATATATAATTTACTAAATTTTACTTTTTTTAAATAAAAAGATATACTTAAAATGAAAAATGTATTTATTTAAGTTAATATTTAAGATGTAATGACTAAATATTAAGGTGTAATTTTAATTAGCTCGATTTTGATTTTAAAATTTTAACTTTGTCTCAATTTGGTCAAATGGATTACAACTCTTAATATTTTTTAATAAAAAATATGTACATAAGTGTCATGTGTTTCAAAATTATAAATTTATCTAACTCAGTAAGTGAACCTCAACTAATTTAGTGATAATTTTTCTTAAAAAATACTAATGATATTATTAATTTGATCAAATTGATGTAAAATCTAAAATTAAGGATCAAAATCAAATCAATTAAAAATGTAAAAACCAAAATAAAAAAAAAAATAAAATATAAGGACATCGGCGTAATTTACCCACAAAATTATCATTTTTCAGTTTTATGATATTTAAAATTGTTTATATGAGTTTGTTGGGCAAGTTCGAGATTGTACTAGCAAGATTTTAAGGATAGCTGGGAGGGATTTTGGATTTGTTTGATTTGTTTCTCATGATATAATTTTAAATTTTTTTTTTTTAATTTTGTTTGGTTTAAATTTTTTGAGTTTTTTTTTTCAAAATCATTTTTTTTTCGTATATATCAATCAGTCACTTTTTCTCTTTTCTCTTTCCTTCTCAATCATTTTTTTTCTCTCACACATATCTACTCAAACTATAATAAAATATCAAATCATTTCAAACTCTCAAACTCCGAAACTATTTTCAAAATATACAACAAAATAAATCAAAAAAATTTCTAAACTCTCTCTCTAAAAAAAAAAATATTTAAAAATTTATTTCAAAACAAAAACCAAACACACCCTTATAGTTTCAAAAACTGAAAAAAATATTTGATGTTATTGGACGTCACGTGGTGTACTACTTACCAATAATAATATATCATGTTTCTATTATTTTTTAATCACGTGGCGTAGCCTAACATCCAGACTATAGATAGGCTTTCAGGCCATTATTCACATCACTACTGTGGTAGCTGGCCTCTTTACTATAAAAATTAGTGCTTTTCTGGTTATTCATATTCATATCACTGCTGTGGCAGCTGGCCTCTTTGCCATAAAAAATTACTTTCCGACGAGGCGTGGAGCTAGCTACT |
| >TdP  GTAGGTCAAGAATGTGGGTTTATATGCATTCTTTTGAAGGCGTTACAGTTTTGCTGTCGCTTTTGGGCTGATTTGCTGTTATTTTTGGGCTGATTTATCATATGTTTAAGCTTCATTTTGAACTAAAAAAAAAACAGTATGATAGGATAGATTAATATGAATAATTTGAGATAGGTCAATCAAATTAATTCTTTCAAATAATCAAAAACATAATCAAAACCAAAACAAATTATCAAAATCAAATATGTCTAACAAAATACATAAGAATACGAAAAGTGTACTGAATTTTTGTATTTTTTTTATTTATATTTTTGGTAATTAGTGAATTTTTTAAAGATTTTTTTTTACAAATATTATACTTTTTAAGTATATTTATGTTAGGTGTATCTAAAAATTTTAAAAAATTGCTTAGAATATTAAAAAAAATTTAGTGAAAACAAAAAAAAAAAAAATGAATTTTTTTTTTTTTCAATTTTCTAAAAAGTGAATCCTGAAAATTCAAACCAAACAAACTAAATTTGTTGTTAGAGTGTTAAATAATTTCTCTATAAGTGTTTGTCAATTTTAATACTTCTTTTAAAGTCTTAAGATTGATATTTAAACTCATTAAATTGGATCAGAATTATTTTTTAAAGTTTTTCGTAGATGGGTTGGATTTGGAAATGTTTCTTCCTGCTCGCCCTAGCATGATTATTAATATATATTATGTACAAGTAAAATTTTACTATAAATACTTCTTATCATATGTATTTTTAACTTATATTATACTTTTAAGTTATACAAATTTTTTTTTCAGTTATAATTAATTTTTTTTCTGTAGTTGCGATCTTATTTTAGTTATGACTCATAATATCTCATGTATTATTTGGTTGACTTTTTTTTTGTTTTTTTGTTTTGACTTTAAAATATGAAATTATGTCATTTTTTTTTAGTTATAAGGTGCATACGATACATACCCTTTAAATTTTTCTCGTATGTTCATATGCACATATTAATATCTGTATATATATAATTTACAAAATTTTACTTTTTTAGAATAAAAAGATATACTTAAAACGAAAAATGTATTTATTTAAGTTAATATTTAAGATGTAATGACTAAATATTATGGTGTAGTTTTAATTTGCTCGATTTTGATTTAAAATTTTTAACTTTGTCTCAATTTGGTCAAATGGATTACAACTGTTAATATTTTTTAATAAAAAAATATATATATAAGTGTTATGTGTCTCAAAATTATGAATTTATCTAACTCAGTAAGTGAACCTCAACTAATTTAGTGATAATTTTTCTTAAAAAACACAAATGATATTATTAATTTGATCAAATTGACGTAAAATATAAAATTAAGGATCAAAATCAAATCAATTAAAAATGTAAAAACTAAAATAAAACAAAAGATAAAATATAAGGACATCCGCTTAATTCACCCACAAAATTATCATTTTTCAGTTTTATAATATTTTAAATTGTTTATATGAGTTTGTTGGGCAAGTTCGAGATTGTACTAGCAAGATTTTAAGGCTAGCTTGGGAGGGATTTTGGGTTTGTTTGATTTGTATATATAATTTTTAATTTTTATTTTTTTTAAATTTTGTTTGGTTTAAATATTTTGAATTTTTTTTTTTCAAAATCACTTTTTTTTTTTTTTCTGTATACATCAATCAGTCACTTTTTCTTTCTTATCTTTTCCTCTCAATCATTTTTTTTTCTCACACACTTCTACTCAAACTATAATAAAATATCAAATCATCCCAAAATCTCAAACTATTTTCAAAATATACAACCAAACAAACCAAAAATATTTATAAACTCTCTCTCAAAAAAAAAAAAAATTTAAAAATTTATCTCAAAACAAAAACCAAACACACCCTTTTAGTTTCAAAAACTGAAAAAAATATTTGGTGTTATTGGACGTCACGTGGCGTACTACTTACCAATAATAATATGTCATGTTTATATTATTTTTTAATCACTTAATATAAAATTATAAGTCTCATTTTTTTTTATTAATTAAAATACTTGTGTATCACGTGCAAAATCAACCAATAATTTCTCAAAAAAAAATCCTAACTTAGGCGTACCCGAACACCTTTTTTTTATTAATTAAAATACTTGTGTATCACGTGCAAAATCAACCAATAATTTCTCAAAAAAAAATCCTAACTTAGGCGTACCCGGACACCCAGACTATAGATAGGCTTTCAGGCCACTATTCACATCACTGCTGTGGTAGCTGGCCTTTTTGCTATAAAAATTAGTGCTTTTCTGGTTATTCATATTCATATCACTGCTGTGGCAGCTGGCCTCTTTGCTATAAAAATTACTTTTCTGACGAGGC |
| >TeP  GTAGGTCAAGAATGTGGGTTTATATGCATTATTTTGAAGGCATTACAGTTTTGCGGTCGTTTTTGGGCTGATTTACTGTTATTTTTGGGCTGATTTATCATATGTTTCAGCTTCATTTTGAACTCAAAAAAAAAAAACAAAAAACAAAAAACAAAATTAGAGCATCAAATAATCAAATAATCTAAGATGATTTAAGGAATTTATATCAGGCAAGACTGAATTTCAATTTGATCTTCAAAAATAACATTATGATAGGATAGATTAATATGAATAATTTGAGATAGGTCAATCAAATTAATTCTTTCAAATAATCAAAAACATAATCAATACAAAAACAAATTATCAAAATCAAATATGTCTAACAAAATACATAAGAATACGAAAAGTGTACTGAATTTTTTTATTTTTTTATTTATATTTTTGGTAATTAGTGAATTTTTTAAAGATTTTTTTTTACAAATATTATACTTTTTAAGTATATTTATGTTAGGTGTATCTAAAAATTTTAAAAAATTGCTTAGAATATTAAAAAAAATTAGTGAAAACAAAAAAAATAAAAAATGAAAAATAAAAAATAAAAAATTTCAATTTTCTAAAAAGTGAATCCTGAAAATTCAACCAAACAAACTAAATTTGTTGTTAGAGTATTAAATAATTTCTCTATAAGTGTTTGTCAATTTTAATACTTCTTTTAAAGTCTTAAGATTGATATTTAAACTCATTAAATTGGATCAGAATTATTTTTTAAAATTTTTCGTAGTTGGGTTGGATTTGGAAATGTTTCTTCCTGCTCGCCCTAGCATGATTATTAATATATATTATGTACATGTAAAATTTTACTATAAATACTTCTTATCGTATGTATTCTTAACTTATATTATACTTTTAAGTTATACAAATTTTTTTTTCAGTTATAATTAATTTTTTTTTTTCTTTAGTTGCGATCTTATTTTAGTTATGACTCATAATATCTCATGTATTATATGGTTGACTTTTTATTTATTTATTTATTTATTTTATATAGTTATGACTTTAAAGTATGAAGTTATGTCATTTTTTTATTTATTTATAAGGTGCATACGATACATACCCTTTAAATTTTTCTCGTATGTACATATGTACATATTAATATCTGTATATATATAATTTACAAAATTTTACTTTTTTAAAATTAAAAGATATACTTAAAATGAAAAATGTATTTATTTAAGTTAATATTTAAGATGTAATGACTAAATATTAAGGTGTAATTTTAATTAGCTCGATTTTGATTTTAAATTTTTAACTTTGTCTCAATTTGGTCAAATGGATTACAACTCTTAATATTTTTTAATAAAAAAATATGTACATAAGTGTCATGTGTCTCAAAATTATGAATTTATCTAACTCAGTAAGTGAACCTCAACTAATTTAGTGATAATTTTTCTTAAAAAACACTAATGATATTGTTAATTTGATCAAATTGACGTAAAATCTAAAATTAAGGATCAAAATCAAATCAATTAAAAATGTAAAAACCAAAATAAAACAAAAGATAAAATATAAAGACATCCGCGTAATTCACCCACAAAATTATCATTTTTCAGTTTTATAATATTTAAAATTGTTTATATGAGTTTGTTGGGCAAGTTCGAGATTGTACTAGCAAGATTTTAAGGCTAGCTTGGGAGGGATTTTGGGTTTGTTTGATTTGTGTCTCATCATATAATTTTTATTTATTTTTTTTAAAATTTTGTTTGGATTAAATATTTTGAATTTTTTTTTTCAAAATCACTTTTTTTTTCTGTATACATCAATCAGTCACTTTTTCTCTCTTATCTTTTCTTCTCAATCATTTTTTTTTCTCACACACATCTACTCAAACTATAATAAAATATCAAATCATCCCAAAATCCCAAACTATTTTCAAAATATACAACCAAATAAACCAAAAAAATTTCTAAACTCTCTCTCAAAAAAAAAAAATTTAAAAATTTATCTCAAAACAAAAACCAAACACACCCTTTTAGTTTCAAAAACTGAAAAAAATATTTGGTGTTATTGGACGTCACGCGGCGTACTACTTACCAATAATAATAATATCGGTTATGCTACAGACCCCCACTGGGGTCGACCCCCACTGAGATGGCAGCCCCTCATTCGTTCAAAACACAAAAAAAAAAAAAAAAAAAAACAGTTTTTTTTTTTTAACCAATGGGAAGCCACCATTCATGACCCCGGTGACTCCCACTGGGGGTCTGAGGCATTTTTCTAATAATATATCATGTTTTTATTATTTTTTAATCACTTAGGCCTAGTTTGGCTACTGCCAAAAAAGTGCACTTATTTCATCTTTTTATCTTTTTTGGGTCACATCAATCAACATCTCCTCTCTACTTTTTCTACACATAATACAAAAAAAGTCAAAAAGTGCACTTATTTAGTCTTCCCAAACTAGGCCTTAATATAAAATTATAAGTCTCATTTTTTTTATTAATTAAAATACTTTTGTGTATCACGTGCAAAATCAACCAATAATTTCTCAAAAAAAAAAATCCTAACCTAGGCGTACCCGAACACCCAGACTGTAGATAGGCTTTCAGGCCATTATTCACATCACTGCTGTGGTAGCTAGCCTCTTTGCTATAAAAATGAGTGCTTTTCTGGTTATTCATATTTATATCACTGCTGTGGCAGCTGGCCTCTTTGCTATAAAAGTTACTTTTCTGACGAGGC |
| >TfP  GTAGGTCAAGAATGTGGGTTTATATGCATTATTTTGAAGGCGTTACAGTTTTGCTGTCGCTTTTGGGCTGATTTGCTGTTATTTTTGGGCTGATTTATCATATGTTTAAGCTTCATTTTGAACTAAAAAAAAAAAAACAGTATGATAGGATAGATTAATATGAATAATTTGAGATAGGTCAATCAAATTAATTCTTTCAAATAAACAAAAACATAATCAAAACCAAAACAAATTATCAAAATCAAATATGTCTAACAAAATACATAAGAATACGAAAAGTGTACTGAATTTTTGTATTTTTTTTATTTATATTTTTGGTAATTAGTGAATTTTTTAAAGATTTTTTTTTACAAATATTATACTTTTTAAGTATATTTATGTTAGGTGTATTTAAAAATTTTAAAAAATTGCTTAGAATATTAAAAAAAATTTAGTGAAAACAAAAAAAAAAAAATGAAAAAAAAAAAATTTCAATTTTCTAAAAAGTGAATCCTGAAAATTCAAACCAAACAAACTAAATTTGTTGTTAGAGTATTAAATAATTTCTCTATAAGTGTTTGTCAATTTTAATACTTCTTTTAAAGTCTTAAGATTGATATTTAAACTCATTAAATTGGATCAGAATTATTTTTTAAAGTTTTTCGTAGATGGGTTGGATTTGGAAATGTTTTTTCCTGCTCGCCCTAGCATGATTATTAATATATATTATGTACACGTAAAATTTTACTATAAATACTTCTTATCATATGTATTTTTAACTTATATTATACTTTTAAGTTATACAAATTTTTTTTTCAGTTATAATTAATTTTTTTTCTGTAGTTGCGATCTTATTTTAGTTATGACTCATAATATCTCATGTATTATATGGTTGACTTTTTTTTTGTTTTTTTGTTTTGACTTTAAAATATGAAATTATGTCATTTTTTTTTAGTTATAAGGTGCATACGATACATACCCTTTAAATTTTTCTCGTATGTTCATATGCACATATTAATATCTGTATATATATAATTTACAAAATTTTACTTTTTTAGAATAAAAAGATATACTTAAAATGAAAAATGTATTTATTTAAGTTAATATTTAAGATGTAATGACTAAATATTAAGGTGTAATTTTAATTTGCTCGATTTTGATTTAAAATTTTTAACTTTGTCTCAATTTGGTCAAATGAATTACAACTCTTAATATTTTTTAATAAAAAAATATATATATAAGTGTCATGTGTCTCAAAATTATGAATTTATCTAACTCAGTAAGTGAACCTCAACTAATTTAGTGATAATTTTTCTTAAAAAACACTAACGATATTATTAATATGATCAAATTGACGTAAAATATAAAATTAAGGATCAAAATCAAATCAATTAAAAATGTAAAAACTAAAATAAAACAAAAGATAAAATATAAGGACATCCGCTTAATTCACCCACAAAATTATCATTTTTCAGTTTTATAATATTTTAAATTGTTTATATGAGTTTGTTGGGCAAGTTCGAGATTGTACTAGCAAGATTTTAAGGCTAGCTTGGGAGGGATTTTGGGTTTGTTTGATTTGTATATATAATTTTTAATTTTTATTTTTTTTAAATTTTGTTTGGTTTAAATATTTTGAATTTTTTTTTTCAAAATCACTTTTTTTTTTTTTTTTTTTTTTTTTTCTGTATACATCAATCAGTCACTTTTTCTTTCTTATCTTTTCCTCTCAATCATTTTTTTTTCTCACACACTTCTACTCAAACTATAATAAAATATCAAATCATCCCAAAATCTCAAACTATTTTCAAAATATACAATCAAACAAACCAAAAATATTTCTAAACTCTCTCTCAAAAAAAAAAAAAATTAAAAATTTATCTCAAAACAAAAACCAAACACACCCTTTTAGTTTCAAAAACTGAAAAAAATATTTAGTGTTATTGGACGTCACGTGACGTACTACTTACCAATAATAATATGTCATGTTTATATTATTTTTTAATCACTTAATATAAAATTATAAGTCTCATTTTTTTTATTAATTTACCCGGACACCCAGACTATAGATAGGCTTTCAGGCCACTATTCACATCACTGCTGTGGTAGCTGGCCTTTTTGCTATAAAAATTAGTGCTTTTCTGGTTATTCATGTTCATATCACTGCTGTGGCAGCTGGCCTCTTTGCTATAAAAATTACTTTTCTGACGAGGC |
| >TgP  GTAGGTCAAGAATGTGGGGTTTATATGCATTATTTTGAGGGCGTTACAGTTTTGCTGTCGTTTTTGGGCTGATTTGCTGTTATTTTTGGGCTGATTTATCATATGTTTAAGCTTCATTTTGAACTCAAAAAAAAAAAAAACAAAAAAAAAAAATTAGAGCATCAAATAATCAATTAATCTAAGATGATTTAAGGAACTTATATCAGGCAAGATTGAATTTCATTTTGATCTTCAAAAATAACATTATGATAGGATAGATTAATATGAATAATTTGAGATATGTCAATCAAATTAATTCTTTCAAATAATCAAAAACATAATCAAAACAAAAACAAATTATCAAAATCAAATATGTCTAACAAAATACATAAGAATACGAAAAGTGTATTGAATTTTTGTATTTTTTGTATTTATATTTTTGGTAATTAGTGAATTTTTTAAAGATTTTTTTTACAAATATTATACTTTTTAAGTATATTTATGTTAGGTGTATCTAAAAATTTTAAAAAATTGCTTAGAATATTAAAAAAAATTTAGTGAAAAAAAAATTTCAATTTTCTAAAAATTGAATCCTGAAAATTCAAACCAAACAAACTAAATTTGTTGTTAGAGTATTAAATAATTTCTCTATAAGTTTTTGTCAATTTTAATACTTCTTTTAAAGTCTTAAGACTGATATTTAAACTCATTAAATTGGATCAGAATTATTTTTTAAAGTTTTTCGTAGATGGGTTGGATTTGAAAATGTTTCTTCCTGCTCGCCCTAGCATGATTATTAATATATATTATGTACATGTAAAATTTTACTATAAATACTTCTTATCGTATGCATTCTTAACTTATATTATACTTTTAAGTTATACAAATTTCTTTTTCAGTTATGATTAATTTTTTTTCTTTAGTTGCGATCTTATTTTAGTTATGACTCATAAGATCTTTGGTTATATCTCATGTATTATTTGGTTATGACTTTTATATATATATATATATAGCTATGACTTTAAAGTATGAAGTTATGTTATTTTTCTTTTAGTTATAAGATGCATACGATACATACCCTTTAAATTTTTCTCGTATGTGCATATGTACATATTAATATCTGTATATATATAATTTACTAAATTTTACTTTTTTTAAATAAAAAGATATACTTAAAATGAAAAATGTATTTATTTAAGTTAATATTTAAGATGTAATGACTAAATATTAAGGTGTAATTTTAATTAGCTCGATTTTGATTTTAAATTTTTAACTTTGTCTCAATTTGGTCAAATGGATTACAACTCTTAATATTTTTTAATAAAAAATATGTACATAAGTGTCATTTGTCTCAAAATTATGAATTTATCTAACTCAGTAAGTGAACCTCAACTAATTTAGTGATAATTTTTCTTAAAAAATACTAATGATATTATTAATTTGATCAAATTGACGTAAAATCTAAAATTAAGGATCAAAATCAAATCAATTAAAAATGTAAAAACCAAAATAAAATAAAAGATAAAATATAAGGACATCGGCGTAATTTACCCACAAAATTATCATTTTTCAGTTTTATGATATTTAAAATTGTTTATGTGAGTTTGTTGGGAAGTTCGAGATTGTACTAGCAAGATTTTAAGGCTAGCTTGGAGGGATTTTGATTTGTTTGATTTGTATCTCATGATATAATTTTTAATTTTTTTTTAAAATTTTGTTTGGTTTAAATTTTTTGAGTTTTTTTTTTCAAAATCAGTTTTTTTTTCGTATACATCAATCAGTCACTTTTTCTCTCTTCTCTTTCCTTCTCAATCATTTTTTTTTCTCTCACACATATCTACTCAAACTATAATAAAATATCAAATCATTCCAAACTCCCAAACTCCGAAACTATTTTCAAAATATACAATAAAACAAACCAAAAAAATTTCTAAACTCTCTCTCAAAAAAAAAAAATATTTAAAAATTTATCTCAAAACAAAAACCAAACACACCCTTTTAGTTTCAAAAACTGAAAAAAATATTTGGTGTTATTGGACGTCACGTGGCGTACTACTTACCAATAATAATATATCATGTTTCTATTATTTTTTAATCACTTAATATAAAATTATAAATCTTATTTTTTTTTACGGTTTTGGTTGGTAGCTCGAAATTTAGGGGCCCTAATGGCCCTAAATTTCCATATAAGGCAAAAAAAAAAAAAACAAGGCAAAAAGAAAAAAGAAAAAAAAAAACAAACTTATCCAGAAAAGGAAATTAGGGCCATTAGGGCCACAGTTTTTAGGGCCACAATTCGTTTTCCTTTTTTTTATTAATTAAAATACTTTTGTGTATCACGTGACAATACCTCAAAATCAACCAATAATTTCTCTCCAAGTCGGGCCGGGTCAGATTTGGATTAAAGATTTTTCTTTTTTTTAAAAAAAAAAATAAAAAAAAAAAAATTCCTAACTTAGGCGTAGCCGAACACCCAGACTATAGATAGGCTTTCAGGCCATTATTCACATCACTACTGTGGCAGCTGGCCTCTTTGCTATAAAATGCTTTTCTGGTTATTCATATTCATATCACTGCTGTGGCAGCTGGCCTCTTTGCCATAAAAAATTACTTTTCTGACGAGGCGTGGAGCTAGCTACT |
| >ThP  GTAGTCAAGAATGTGGGTTTATATGCATTATTTTGAAGGCGTTACAGTTTTGCTGTCGCTTTTGGGCTGATTTGCTGTTATTTTTGGGCTGATTTATCATATGTTTAAGCTTCATTTTGAACTAAAAAAAAAAAAAAAAAAAACATTAGGATAGGATAGATTAATATGAATAATTTGAGATAGGTCAATCAAATTAATTCTTTCAAATAATCAAAAACATAATCAAAACCAAAACAAATTATCAAAATCAAATATGTCTAACAAAATACATAAGAATACGAAAAGTGTACTGAATTTTTGTATTTTTTTATTTATATTTTTGGTAATTAGTGAATTTTTTAAAGATTTTTTTTTTTACAAATATTATACTTTTTAAGTATATTTATGTTAGGTGTATCTAAAAATTTTAAAAAATTGCTTAGAATATTAAAAAAATTTAGTGAAAAAAAAAAATGAAAAAAAAAAAATTTCAATTTTCTAAAAAGTGAATTCTGAAAATTCAAACCAAACAAACTAAATTTGTTGTTAGAGTATTAAATAATTTATCTATAAGTGTTTGTCAATTCTAATACTTCTTTTAAAGTCTTAAGATTGATATTTAAACTCATTAAATTGGATCAGAATTATTTTTTAAAGTTTTTCCTAGATGGGTTGAATTTGGAAATGTTTCTTCCTGCTCGCCCAAGCATGATTATTAATATATATTATGTACACGTAAAATTTTACTATAAGTACTTCTTATCATATGTATTTTTAACTTATATTATACTTTTAAGTTATACAATTTTTTTTTTCAGTTATAATTAATTTTTTTTCTGTAGTTGCGATCTTATTTTAGTTATGACTCATAATATCTCATGTATTATTTGGTTGACTTTTTTTTTTTTTTTTTGTTTTGACTTTAAAATATGAAATTATGTCATTTTTTTTTAGTTATAAGGTGCATACGATACATACCCTTTAAATTTTTCTCGTATGTACATATGTACATATTAAATCTGTATATATATAATTTACAAAATTTTACTTTTTTAGAATAAAAAGATATACTTAAAATGAAAAACGTATTTATTTAAGTTAATATTTAAGATGTAATGACTAAATATTAAGGTGTAATTTTAATTAGCTCGATTTTGATTTTAAATTTTTAACTTTGTCTCAATTTGGTCAAATGGATTACAACTCTAAATATTTTTTAATAAAAAAATATGTATATAAGTGTCATGTGTCTCAAAATTATGAATTTATCTAACTCAGTAAGTGAACCTCAACTAATTTATTAGTGATAATTTTTCTTAAAAAACACTAATGATATTATTAATTTGATCAAATTGACGTAAAATCCAAAATTAAGGATCAAAATCAAATCAATTAAAAATGTAAAAACTAAAATAAAACAAAAGATAAAATATAAGGACATCCGCTTAATTCACCCACAAAATTATCGTTTTTTAGTTTTATAATATTTTAAGTTGTTTATATGAGTTTGTTGCGCAAGTTCGAGATTGTACTAGCAAGATTTTAAGGCTAGCTTGGGAGGGATTTTGGGTTTGTTTGATTTGTATCTCATGATATAGTTTTTAATTTTTATTTTTTTAAATTTTGTTTGGTTTAAATATTTTGAATTTTTTTTTTTCAAAATCACTTTTTTTTCTGTATACCTCAATCAGTCACTTTTTTTTTCTTATCTTTTCCTCTCAATCATTTTTTTTTTTCTCACACACTTCTACTCAAACTATAATAAAATATCAAATCATCCCAAAATCTCAAACTATTTTCAAAATATACAACCAAACAAACCAAAAATATTTCTAAACTCTATCTCAAAAAAAAAAAAAATTTAAAAATTTATTTCAAAACAAACACCAAACACACCCTTTTAGTTTCAAAAACTGAAAAAAATATTTACTGTTATTGGACGTCACGTGGCGTACTACTTACCAATAATAATGTCATGTTTCTATTATTTTCTAATCACTTAATATAAAGTTATAAGTCTCATTTTTTTTTTATTAATTAAAATACTTGTGTATCACGTGCAAAATCAACCAATAATTGCTCCAAAAAAATATAGATAGGCTTTCAGGCCATTATTCACATCACTGCTGTGGTAGCTGGCCTCTTTGCTATAAAAATTAGTGCTTTTCTGGTTATTCATATTCATATCACTGCTGTGGCAGCTGGCCTCTTTGCTATAAAAATTACTTTTCTGACGAGGC |
| >Ti1P  GTAGGTCAGAATGTGGGTTTATATGCATTATTTTGAGGGCGTTACAGTTTTGCTGTCGTTTTTGGGCTGATTTGCTGTTATTTTTGGGCTGATTTATCATATGTTTAAGCTTCATTTTGAACTCCAAAAAAAAAAAAAAAACAAAATTAGAGCATCAAATAATCAATTAATCTAAGATGATTTAAGGAACTTATATCAGGCAAGATTGAATTTCATTTTGATCTTCAAAAATAACATTATGATAGGATAGATTAATATGAATAATTTGAGATATGTCAATCAAATTAATTCTTTCAAATAATCAAAAACATAATCAAAACAAAAACAAATTATCAAAATCAAATATGTCTAACAAAATACATAAGAATACGAAAAGTGTACTAAATTTTTGTATTTTTTGTATTTATATTTTTGGTAATTAGTGAATTTTTTAAAGATTTTTTTTTACAAATATTATACTCTTTAAGTATATTTATGTTAGGTGTATCTAAAAATTTTTAAAAATTGCTTAGAATATTAAAAAAAATTTAGTGAACACAAAAAAAAAAAAAAAAAAAAAAAAATTTTTTTTTTAATTTTCTAAAAAGTGAATTCTGAAAATTCAAACCAAACAAACTAAATTTGTTGTTAGAGTATTAAATAATTTCTCTATAAGTGTTTGCCAATTTTAATACTTCTTTTAAAGTCTTAAGACTGATATTTAACCTCATTAAATTGGATCAGAATTATTTTTTAAAGTTTTTCTTAAATGGGTTGGATTTGGAAATGTTTCTTCTTGCTCGCCCTAGCATGATTATTAATATATATTATGTACATGTAAAATTTTATTATAAATACTTCTTATCGTATGCATTCTTAACTTATATTATACTTTTAAGTTATAAAATTTTTTTTTTCAGTTGTGATTAATTTTTTTTCTTTAGTTGCGATCTTATTTTAGTTATGACTCATAAGATCTTTGGTTATATCTCGTGTATTATTTGGTTATGACTTTATATATATATATATATATATAGCTATGACTTTAAAGAATGAAGTTATGTCATTTTTCTTTTAGTTATAAGGTGCATACGATACATACCCTTTAAATTTTTCTTGTATGTGCATATGTACATATTAATATCTGTATATATATAATTTACTAAATTTTACTTTTTTTAAATAAAAAGATATACTTAAAATGAAAAATGTATTTATTTAAGTTAATATTTAAGATGTAATGACTAAATATTAAGGTGTAATTTTAATTAGCTCGATTTTGATTTTAAAATTTTAACTTTGTCTCAATTTGGTCAAATGGATTACAACTCTTAATATTTTTTAATAAAAAATATGTACATAAGTGTCATGTGTCTCAAAATTATAAATTTATCTAACTCAGTAAGTGAACCTCAACTAATTTAGTGATAATTTTTCTTAAAAAATACTAATGATATTATTAATTTGATCAAATTGACGTAAAATCTAAAATTAAGGATCAAAATCAAATCAATTAAAAATGTAAAAACCAAAATAAAAAAAAAAAAAATATAAGGACATCGGCGTAATTTACCCACAAAATTATCATTTTTCAGTTTTATGATATTTAAAATTGTTTATATGAGTTTGTTGGGCAAGTTCGAGATTGTACTAGCAAGATTTTAAGGATAGCTGGGAGGGATTTTGGATTTGTTTGATTTGTTTCTCATGATATAATTTTTAAATTTTTTTTTTTAATTTTGTTTGGTTTAAATTTTTTGAGTTTTTTTGTTAAAATCATTTTTTTTTTTTCGTATACATCAATCAGTCACTTTTTCTCTTTTCTCTTTCCTTCTCAATCATTTTTTTTCTCTCACACATATCTACTCAAACTATAATAAAATATCAAATCATTCCAAACTCTCAAACTCCGAAACTATTTTCAAAATATACAACAAAATAAATCAAAAAAATTTCTAAACTCTCTCTCAAAAAAAAAAATATTTAAAAATTTATCTCAAAATAAAAACCAAATACACCCTTATAGTTTCAAAAACTGAAAAAAATATTTGATGTTATTGGACGTCACGTGGCATACTACTTACCAATAATAATATCAACAATGATTCATCCCACTAAAAATATCATCACTAATAGCACTAATACATTAAAAAAGTTAAAAAAAAAAAAAAATACACAACTCCAAGACCAAAACATCACATCATTAATGACGGGACCCACTGCCACCTAATTAGTGGAAGCACTAATTAATGCTCCCAAGCATTTTTCTAATAATATATCATGTTTCTATTATTTTTTAATCACGTGGCGTAGCCTAACATCCAGACTATAGATAGGCTTTCAGGCATTATTCACATCACTACTGTGGTAGCTGGCCTCTTTACTATAAAAATTAGTGCTTTTCTGGTTATTCATATTCATATCACTGCTGTGGCAGCTGGCCTCTTTGCCATAAAAAATTACTTTCCGACGAGGCGTGGAGCTAGCTACT |
| >Ti2P  GTAGGTCAGAATGTGGGTTTATATGCATTATTTTGAGGGCGTTACAGTTTTGCTGTCGTTTTTGGGCTGATTTGCTGTTATTTTTGGGCTGATTTATCATATGTTTAAGCTTCATTTTGAACTCCAAAAAAAAAAAAAACAAAAAAAAAACAAAATTAGAGCATCAAATAATCAATTAATCTAAGATGATTTAAGGAACTTATATCAGGCAAGATTGAATTTCATTTTGATCTTCAAAAATAACATTATGATAGGATAGATTAATATGAATAATTTGAGATATGTCAATCAAATTAATTCTTTCAAATAATCAAAAACATAATCAAAACAAAAACAAATTATCAAAATCAAATATGTCTAACAAAATACATAAGAATACGAAAAGTGTACTAAATTTTTGTATTTTTTGTATTTATATTTTTGGTAATTAGTGAATTTTTTAAAGATTTTTTTTTACAAATATTATACTCTTTAAGTATATTTATGTTAGGTGTATCTAAAAATTGCTTAGAATATTAAAAAAAATTTAGTGAACACAAAAAAAAAAAAAAAAAAATTTTTTTTTTTTTTAAATTTTCTAAAAAGTGAATTCTGAAAATTCAAACCAAACAAACTAAATTTGTTGTTAGAGTATTAAATAATTTCTCTATAAGTGTTTGTCAATTTTAATACTTCTTTTAAAGTCTTAAGACTGATATTTAAACTCATTAAATTGGATCAGAATTATTTTTTAAAGTTTTTCTTAGATGGGTTGGATTTGGAAATGTTTCTTCTTGCTCGCCCTAGCATGATTATTAATATATATTATGTACATGTAAAATTTTATTATAAATACTTCTTATCGTATGCATTCTTAACTTATATTACACTTTTAAGTTATAAATTTTTTTTTTTCAGTTGTGATTAATTTTTTTTCTTTAGTTGCGATCTTATTTTAGTTATGACTCATAAGATCTTTGGTTATATCTCGTGTATTATTTGGTTATGACTTTATATATATATATATACAACAATGATTCATCCCACTAAAAACATGACCACTAATAGCACTAATGCATTAAAAAAGTAAAAAAAAAAAAAAAAAATACAACTCAAAGACCAAAACATCACATCATTAATGACGGGACCCACTGCCACCTAATTAGTGGGAGCACTAATTAGTGCTCCCAAGCATTTTTCATATATATATAGCTATGACTTTAAAGAATGAAGTTATGTCATTTTTCTTTTAGTTATAAGGTGCATACGATACATACCCTTTAAATTTTTCTTGTATGTGCATATGTACATATTAATATCTGTATATATATAATTTACTAAATTTTACTTTTTTTAAATAAAAAGATATACTTAAAATGAAAAATGTATTTATTTAAGTTAATATTTAAGATGTAATGACTAAATATTAAGGTGTAATTTTAATTAGCTCGATTTTGATTTTAAAATTTTAACTTTGTCTCAATTTGGTCAAATAGATTACAACTCTTAATATTTTTTAATAAAAAATATGTACATAAGTGTCATGTGTCTCAAAATTATAAATTTATCTAACTCAGTAAGTGAACCTCAACTAATTTAGTGATAATTTTTCTTAAAAAATACTAATGATATTATTAATTTGATCAAATTGACGTAAAATCTAAAATTAAGGATCAAAATCAAATCAATTAAAAATGTAAAAACCAAAATAAAAAAAAAAAAAAATATAAGGACATCGGCGTAATTTACCCACAAAATTATCATTTTTCAGTTTTATGATATTTAAAATTGTTTATATGAGTTTGTTGGGCAAGTTCGAGATTGTACTAGCAAGATTTTAAGGATAGCTGGGAGGGATTTTGGATTTGTTTGATTTGTTTCTCATGATATAATTTTTAAATTTTTTTTTTTTAATTTTGTTTGGTTTAAATTTTTTGAGTTTTTTTTTTAAAATCATTTTTTTTTTTCGTATACATCAATCAGTCACTTTTTCTCTTTTCTTTTTCCTTCTCAATCATTTTTTTTCTCTCACACATATCTACTCAAACTATAATAAAATATCAAATCATTCCAAACTCTCAAACTCCGAAACTATTTTCAAAATATACAACAAAATAAATCAAAAAAATTTCTAAACTCTCTCTCAAAAAAAAAAATATTTAAAAATTTATCTCAAAATAAAAACCAAATACACCCTTATAGTTTCAAAAACTGAAAAAAATATTTGATGTTATTGGACGTCACGTGGCGTACTACTTACCAATAATAATATCAACAATGATTCATCCCACTAAAAATATCACCACTAATAGCACTAATACATTAAAAAAGTTAAAAAAAAAAAAAAAACACAACTCCAAGACCAAAACATCACATCATTAATGACGGAACCCACTGCCACCTAATTAGTGGAAGCACTAATTAATGCTCCCAAGTATTTTTCTAATAATATATCATGTTTCTATTATTTTTTAATCACGTGGCGTAGCCTAACATCCAGACTATAGATAGGCTTTCAGGCATTATTCACATCACTACTGTGGTAGCTGGCCTCTTTACTATAAAAATTAGTGCTTTTCTGGTTATTCATATTCATATCACTGCTGTGGCAGCTGGCCTCTTTGCCATAAAAAATTACTTTCCGACGAGGCGTGGAGCTAGCTACT |

**Table S6.** Comparison promoter sequences of different *TCS1* alleles.

|  | TaP | TbP | Tc1P | Tc2P | TdP | TeP | TfP | TgP | ThP | Ti1P | Ti2PP |
| --- | --- | --- | --- | --- | --- | --- | --- | --- | --- | --- | --- |
| TaP | - | - | - | - | - | - | - | - | - | - | - |
| TbP | 72.05 | - | - | - | - | - | - | - | - | - | - |
| Tc1P | 71.07 | 81.19 | - | - | - | - | - | - | - | - | - |
| Tc2P | 70.89 | 87.28 | 84.55 | - | - | - | - | - | - | - | - |
| TdP | 80.49 | 68.09 | 71.47 | 72.92 | - | - | - | - | - | - | - |
| TeP | 78.35 | 72.57 | 69.34 | 79.90 | 67.92 | - | - | - | - | - | - |
| TfP | 75.19 | 74.74 | 73.05 | 72.73 | 90.33 | 80.16 | - | - | - | - | - |
| TgP | 72.46 | 80.86 | 77.09 | 83.68 | 67.75 | 70.41 | 72.32 | - | - | - | - |
| ThP | 81.70 | 74.63 | 71.79 | 66.74 | 80.94 | 76.12 | 77.11 | 73.68 | - | - | - |
| Ti1P | 71.12 | 81.76 | 83.40 | 84.77 | 72.23 | 73.28 | 71.32 | 77.49 | 67.96 | - | - |
| Ti2P | 69.38 | 80.33 | 75.01 | 87.02 | 72.70 | 69.91 | 68.67 | 77.70 | 74.09 | 97.13 | - |
